# Lipid-lowering drug targets and Parkinson's disease: A sex-specific Mendelian randomization study

**DOI:** 10.3389/fneur.2022.940118

**Published:** 2022-09-01

**Authors:** Yangfan Zhao, Sarah A. Gagliano Taliun

**Affiliations:** ^1^Faculty of Medicine, Université de Montréal, Montréal, QC, Canada; ^2^Department of Medicine, Department of Neurosciences, Faculty of Medicine, Université de Montréal, Montréal, QC, Canada; ^3^Montreal Heart Institute, Montréal, QC, Canada

**Keywords:** Mendelian randomization (MR), sex-specific, Parkinson's disease, low-density lipoprotein cholesterol (LDL-C), risk factor

## Abstract

Parkinson's disease (PD) affects millions of individuals worldwide, and it is the second most common late-onset neurodegenerative disorder. There is no cure and current treatments only alleviate symptoms. Modifiable risk factors have been explored as possible options for decreasing risk or developing drug targets to treat PD, including low-density lipoprotein cholesterol (LDL-C). There is evidence of sex differences for cholesterol levels as well as for PD risk. Genetic datasets of increasing size are permitting association analyses with increased power, including sex-stratified analyses. These association results empower Mendelian randomization (MR) studies, which, given certain assumptions, test whether there is a causal relationship between the risk factor and the outcome using genetic instruments. Sex-specific causal inference approaches could highlight sex-specific effects that may otherwise be masked by sex-agnostic approaches. We conducted a sex-specific two-sample cis-MR analysis based on genetic variants in LDL-C target encoding genes to assess the impact of lipid-lowering drug targets on PD risk. To complement the cis-MR analysis, we also conducted a sex-specific standard MR analysis (using genome-wide independent variants). We did not find evidence of a causal relationship between LDL-C levels and PD risk in females [OR (95% CI) = 1.01 (0.60, 1.69), IVW random-effects] or males [OR (95% CI) = 0.93 (0.55, 1.56)]. The sex-specific standard MR analysis also supported this conclusion. We encourage future work assessing sex-specific effects using causal inference techniques to better understand factors that may contribute to complex disease risk differently between the sexes.

## Introduction

Late-onset Parkinson's disease (PD) is a multifactorial neurodegenerative disorder that affects millions of individuals worldwide (1, 2). It is primarily marked by the death of dopaminergic (DAergic) neurons in the substantia nigra (3) and subsequent loss of dopaminergic innervation to the striatum (4). Pathologically, it is characterized by the presence of Lewy bodies, neuronal inclusions containing α-synuclein (3). There is no cure for PD, and medications only alleviate symptoms (5). The most common treatment option is the medication levodopa (L-dopa), a precursor to dopamine. However, response to L-dopa diminishes as the disease progresses, essentially leaving no further potent treatment as options for individuals affected by PD (3).

The onset and progression of PD is a result of both genetic and non-genetic factors (1). Large-scale genome-wide association studies (GWAS) have been undertaken, identifying around 80 genomic loci contributing to late-onset PD risk, and it is estimated that genetic factors could contribute 20–30% of the observed heritability (6–8). Additionally, there is evidence of sex differences in PD, with males being ~1.5× more likely to develop the disease compared to females (9), and certain symptoms being more common in one sex compared to the other (10–17). Notably, there are also sex differences in terms of disease progression (18) and response to current treatment (19–21). The role of genetics on these sex differences is only beginning to be explored (22). Finding new treatment options, including treatment options that account for differences between females and males, will be key to improving the quality of life of individuals living with PD.

Many modifiable risk factors as well as drug re-purposing options have been explored as options for decreasing risk, delaying onset, or as novel treatments for PD, including circulating levels of low-density lipoprotein cholesterol (LDL-C), which will be the focus of this work. Circulating lipid and lipoprotein metabolism and their respective levels differ between the sexes as a result of multiple variables including genetics, fat distribution, hormones, and environmental factors (23). Work has been done assessing the effect of LDL-C on PD risk, but most studies are sex-agnostic, and the role of LDL-C on PD risk remains unclear.

Circulating LDL-C levels have a plausible biological influence on PD risk, with several hypotheses to support either an inverse or a positive relationship between this risk factor and PD. The brain is the most cholesterol-rich organ in the body, containing around 20% of the total cholesterol in the body (24). Cholesterol is essential to brain health, being not only a component of cellular membranes and myelin sheaths (24) but also playing essential roles in synaptogenesis, dendrite formation, and axonal guidance (25–27). It is important to note that nearly all (>95%) of the cholesterol in the brain is synthesized *de novo* since the blood-brain-barrier (BBB) prevents its uptake from the periphery (28). However, its metabolites, oxysterols, can cross the BBB (29).

In light of this, 27-hydroxycholesterol (27-OHC, derived in majority from the periphery) and 24-hydroxycholesterol (24S-OHC, derived in majority from the brain) (30) have both been studied in the context of PD pathogenesis and are key factors in proposed pathways for an increase in PD risk caused by hypercholesterolemia. 27-OHC, which increases in the brain in situations of hypercholesterolemia (31) or oxidative stress (32), has been shown to increase α-synuclein expression (33, 34) and cell apoptosis (34) in neuron cell culture. 27-OHC also decreased the expression of tyrosine hydroxylase, the rate-limiting step in DA synthesis (33, 34). 24S-OHC, while also capable of inducing cell death (35, 36), increased the expression of tyrosine hydroxylase and decreased that of α-synuclein in neuron cell culture (34). *In vivo* findings include an increase of 27-OHC and 24S-OHC in the cerebrospinal fluid (CSF) of a proportion of PD patients, with 24S-OHC levels correlating with disease duration. This was hypothesized to be caused by neurodegenerative processes and, in the case of 27-OHC, possible BBB defects (37). Cholesterol and oxysterols, including 27-OHC and 24S-OHC, have also been found in one study to be increased in the cortex of PD patients (38); however, other studies reported no significant changes in cholesterol levels in the substantia nigra (39) and putamen (40) of PD patients.

High plasma cholesterol by itself also has links with PD pathogenesis, as shown by the increase of neuroinflammation and oxidative stress in the brains of animals fed high-fat diets (41, 42). High-fat diets also exacerbate striatal DA depletion in animal models of PD (43–45). Moreover, cholesterol-enriched medium also promoted α-synuclein aggregation in α-synuclein-transfected neuronal cells (46).

Conversely, there are also hypotheses for a protective effect of high serum cholesterol on PD risk. These include serum cholesterol's influence on peripheral Coenzyme Q10 levels (47), a mitochondrial electron acceptor and antioxidant that has shown promise in mitigating PD progression in animal models of the disease (48, 49). Cholesterol and Coenzyme Q10 share a biosynthetic pathway, and the majority of circulating Coenzyme Q10 are incorporated into VLDL-C and LDL-C. As such, serum cholesterol is one of the most important determinants of serum Coenzyme Q10 levels. In addition, cholesterol can be incorporated with ferrous iron by neuromelanin, a mechanism that can prevent neurodegeneration (29).

Mendelian randomization (MR) is an epidemiological approach which, under specific assumptions, intends to estimate causal effects between an exposure and outcome of interest. A recent sex-agnostic cis-MR study (using genetic variants within lipid-lowering drug targets) did not find evidence of genetically-determined LDL-C influencing PD risk (50). However, sex-specific MR could move the field closer toward personalized medicine by highlighting sex-specific effects that may otherwise be masked by sex-agnostic approaches. Genetic datasets of increasing size through the emergence of large biobanks and meta-analyses are beginning to permit sex-stratified analyses with adequate statistical power. Below we review the literature on the relationship between LDL-C and PD risk, from both observational and MR studies, and then we assess the possible sex-specific impact of genetically-determined LDL-C levels on PD risk through sex-specific MR.

Most observational studies conducted in the early 2000s assessing the relationship between circulating LDL cholesterol (LDL-C) and/or total cholesterol (TC) levels with PD risk suggest an inverse relationship (51–54), although there were two cohort studies that found, respectively, no evidence of an association between TC and PD risk (55) and evidence of a positive association (56).

More recently, larger studies, with case-control and/or cohort designs, have suggested either no significant association (57) or a negative association (58–63) between cholesterol (LDL-C and/or TC) and PD risk. A meta-analysis by Gudala et al. (64), which included 4 case-control and 4 cohort studies found no significant association between TC and PD [pooled risk ratio (RR) with random-effects model (95% CI) 0.87 (0.67, 1.13), *p* = 0.41]. A 2020 meta-analysis by Fu et al. (65), assessing 13 case-control studies and 8 cohort studies, found a negative association between PD risk and serum levels for both TC [Standardized Mean Difference (SMD) = −0.21 (−0.33, −0.10), *p* = 0.0002 and RR 0.86 (0.77, 0.97), *p* = 0.01] and LDL-C [SMD = −0.26 (−0.43, −0.07), *p* = 0.006 and RR 0.76 (0.59, 0.97), *p* = 0.03]. However, both associations had high heterogeneity (I^2^ ≥ 35%), warranting caution in interpretation. Jiang et al.'s 2020 meta-analysis (66) also reported a negative association between LDL-C and PD risk; an inverse relationship was present in both a high *vs*. low LDL-C comparison [5 cohort studies with 2,406 PD cases: random-effects pooled RR 0.73 (0.57, 0.93), *p* = 0.079] and a dose-response analysis [4 cohort studies with 2,300 cases: 1 mmol/L increase in LDL-C with RR 0.93 (0.88, 0.99), *p* = 0.084]. However, the authors found no significant association between TC and PD risk.

Interpretation of observational studies remains difficult as it is not possible to draw conclusions on causality through such a design. Beside the limitations posed by possible confounders, there is also reverse causation bias [as pointed out by Scigliano et al. (67)] since the prodromal phase of PD, which occurs over an undefined period of several years (68), can lead to lifestyle changes as well as lowered efficiency of the autonomous nervous system (69, 70) that can influence LDL-C and TC levels. Additionally, cohort studies suffer from smaller sample sizes due to the relatively low incidence of PD. Finally, studies are limited by differing definitions of PD cases.

In recent years, MR has provided an exciting new avenue to elucidating the relationship between genetically-determined serum cholesterol levels and PD risk by circumventing several limitations of traditional observational studies. MR studies tend to be less vulnerable to reverse causation bias and confounders due to their use of genetic variants as instrumental variables to proxy for the exposure (e.g., LDL-C levels), since genotypes are assigned at birth, stable throughout the lifespan, and unaltered by the exposure of interest or other factors. However, the validity of the causal inference depends on at least three core assumptions being met. The first is that the genetic variants used to proxy for the exposure are robustly associated with the exposure. The second assumption is that there is no confounding (measured or unmeasured) of the genetic variants with the outcome (e.g., PD), and the third is that the selected variants influence the outcome only through the exposure. The last two assumptions cannot be statistically evaluated, and thus sensitivity analyses must be performed to assess whether the assumptions are likely to have been violated (71).

Sex-agnostic MR has been previously used to test whether a causal relationship exists between genetically-determined cholesterol levels and PD risk (50, 72–74). In brief, three of the four studies so far found no significant association between LDL-C and PD risk (50, 72, 74), while one found a negative association between PD development and LDL-C as well as TC (73). Results from the Williams et al. study are described below, and details on the other studies are in Supplementary material. Williams et al. (50) examined the relationship between circulating cholesterol levels and PD risk with two-sample cis-MR (either using adjusted estimates from correlated variants within gene regions of four lipid-lowering drug classes or based on uncorrelated variants within those gene regions). “Standard” (genome-wide) two-sample MR was also conducted using 52 lipid-associated uncorrelated variants as instruments drawn from three GWAS of LDL-C, triglycerides, and ApoB (sample size range = 14,004–295,826). Variant-PD association estimates were determined using summary data from the International Parkinson's Disease Genomics Consortium and 23andMe (total *N* = 37,688 cases and 981,372 controls). The cis-MR and standard genome-wide models found no significant association between LDL-C and PD risk. None of the exposures (LDL-C, triglycerides, and ApoB) were significantly associated with PD risk in the standard MR analyses, but there was a suggestive protective effect for PD risk by the lowering of triglycerides and ApoB by ApoA5 and/or ApoC3 modulation in the cis-MR models. Williams et al. concluded that their results suggest that peripheral lipid concentrations may play no etiological role in PD risk.

Despite evidence of differences between males and females for cholesterol levels and PD in terms of several factors, including genetics, metabolism, clinical manifestation, and drug response (19, 75–80), which we summarize in Supplementary Table 2, MR analyses assessing this relationship have been conducted in a sex-agnostic manner. We thus aimed to assess the potential role of genetically-predicted LDL-C on PD risk in a sex-specific MR framework to potentially uncover effects masked by sex-agnostic approaches.

## Methods

The methods describing how the literature review of the possible role of cholesterol levels on PD risk was carried out is described in the Supplementary material, along with a more detailed overview of published sex-agnostic MR studies examining this topic.

We followed the Strengthening the Reporting of Observational Studies in Epidemiology using Mendelian Randomization (STROBE-MR) guidelines for reporting MR studies (Supplementary Table 1) (81, 82). Literature review of the possible role of cholesterol levels on Parkinson's disease risk: additional sex-agnostic Mendelian randomization studies.

### Power calculations

Power calculations for the female-only and male-only analyses were performed using the https://shiny.cnsgenomics.com/mRnd/, which is based on the approach described by Burgess (83).

### Summary statistics for effects of instrumental variables on exposure and outcome

We obtained sex-specific variant-trait association results for the genetic instruments for the exposure (circulating levels of LDL-C) from sex-stratified summary statistics for LDL-C that was directly measured (rather than estimated from an equation) from “round 2” of the UK Biobank analysis conducted by the Neale lab (http://www.nealelab.is/uk-biobank), visualized in Supplementary Figure 1A. To our knowledge, sex-stratified summary statistics are not currently available for related traits triglycerides or ApoB [which were also used as exposures in the Williams et al. study in addition to LDL-C (50)], and thus, we focused only on LDL-C. The UK Biobank resource has been described in detail (84). Briefly, the biobank is a population-based prospective cohort study of around half a million individuals (roughly 51% female) aged 40–69 years at recruitment. Participants were recruited across 22 recruitment centers in the United Kingdom between 2006 and 2010. LDL-C was measured in mmol/L through blood chemistry analysis by enzymatic protective selection analysis on a Beckman Coulter AU5800 at the initial assessment visit at which participants were recruited and consent given. The female-specific and male-specific association analyses conducted by the Neale lab assessed 184,689 and 158,932 individuals, respectively, and tested the inverse variant rank normalized values from UK Biobank field 30780 for association genome-wide (autosomes and chromosome X) on European-ancestry participants. Age, age∧2, and the first twenty principal components for genetic ancestry were included as covariates in the models.

For the association results for the outcome (PD), we downloaded sex-stratified summary statistics (autosomes only) from the analysis conducted by Blauwendraat et al. (80) from the International Parkinson's Disease Genomics Consortium website (https://pdgenetics.org/), visualized in Supplementary Figure 1B. For most cohorts in this meta-analysis, PD ascertainment was through clinical assessment, and control participants were excluded if they had any known neurological diseases. Sex-stratified analyses were based on genetic sex. To ensure independence of study individuals between the PD (outcome) and the LDL-C (exposure) sex-stratified summary statistics, and thus permitting a two-sample MR study design, we used the version of the PD summary statistics in which the UK Biobank individuals were removed. 23andMe individuals are not included in the publicly available results. Details of the individual cohorts included in the analysis can be found in Supplementary Table 3. In brief, the non-UK Biobank association results were derived from a meta-analysis of up to 17 European-ancestry case-control studies (12,054 male cases, 19,336 male controls, 7,384 female cases, and 20,330 female controls) in which the following covariates were included: age at onset for cases and age of last examination for controls, the first five principal components of genetic ancestry, and dataset origin. Bi-allelic variants with a minor allele frequency > 1% that had a meta-analysis heterogeneity statistic (I^2^) of <80% were retained. A total of 19 genome-wide significant regions but no sex-specific effects were observed. The authors reported a high genetic correlation between the male and female analyses (rg = 0.877), and very similar heritability estimates between male and female PD cases (~20%).

### Sex-specific cis-Mendelian randomization

We conducted sex-specific (female-only and male-only) two-sample cis-MR with uncorrelated genetic variants to assess the relationship between genetically-predicted LDL-C levels on late-onset PD risk, and visualized results in R (version 4.0.5) (85) using the package *TwoSampleMR*, version 0.5.6 (86). The packages *MendelianRandomization*, version 0.5.1 (87) and *MR-PRESSO* version 1.0 (88) were used to obtain the ${\text{I}}_{\text{GX}}^{2}$ from MR-Egger regression (a statistic ranging between 0 and 1 assessing whether genetic variant-exposure associations are sufficiently heterogeneous) and the MR-PRESSO outlier test, respectively. In cases where the residual standard error in the MR-Egger regression was less than one, we report the *MendelianRandomization* implementation of the *p*-value in which the residual standard error is set at 1 and a z-distribution is used to obtain the corresponding *p*-value, instead of the more conservative *TwoSampleMR* implementation using the residual standard error set at 1 and a t-distribution.

As genetic instruments, we started with the same genetic variants that were used in the Williams et al. (50) sex-agnostic cis-MR analysis of uncorrelated variants for LDL-lowering gene targets weighted by LDL-C. The selected variants fall within ± 100 kilobases of the gene start/stop coordinates (*APOB*- 2 variants, *HMGCR*- 1 variant, *NPC1L1*- 1 variant, *PCSK9*- 2 variants), have a significant (*p* < 5 × 10^−8^) GWAS association with LDL-C and are independent from each other (pairwise r^2^ < 0.001). We verified that the selected variants also had genome-wide significant associations with LDL-C (*p* < 5 × 10^−8^) in the sex-stratified UK Biobank LDL-C summary statistics. Since one of the variants (rs2073547) was not available in the exposure association statistics, we used the LDlink resource (89) to select the non-palindromic rs17725246 as a proxy, given the high linkage disequilibrium between these two variants of r^2^ = 0.98 (based on European genetic ancestry samples in the 1000 Genomes Project). We obtained the variant-exposure and variant-outcome effect sizes from the sex-stratified association results for LDL-C and PD, respectively.

The study protocol was not pre-registered, but we determined in advance the MR approach for the primary analysis. Specifically, in the primary analysis we used the inverse-variance weighted (IVW) method with random effects, and we conducted MR-Egger, weighted median, simple mode, and weighted mode as sensitivity analyses to assess consistency of results. Although horizontal pleiotropy is minimized in a cis-MR analysis as only genetic variants within the vicinity of genes encoding drug targets of interest are included, we still ran tests to detect the presence of pleiotropy as an additional check, specifically the MR-Egger intercept test and the MR-PRESSO (88) outlier test. To assess directionality (whether the direction of effect is from LDL-C to PD or if the opposite is a possibility), we used Steiger filtering (90) as implemented in the directionality_test function of the *TwoSampleMR* R package. Results were expressed as PD risk per standard deviation lower LDL-C.

### Sex-specific standard Mendelian randomization analysis

To complement the cis-MR analyses, we also performed sex-specific standard MR using as genetic instruments genome-wide independent variants robustly associated with LDL-C levels from female-specific and male-specific GWAS conducted in the UK Biobank. For genetic instruments, we started with the independent LDL-associated variants used in the standard non-sex-specific MR analysis listed in Supplementary Table 6 of Williams et al. (50). For the female-only analysis, we retained instruments if they were genome-wide significant (*p* < 5 × 10^−8^) in the female-specific LDL-C GWAS. Similarly, for the male-only analysis, we retained instruments if they were genome-wide significant in the male-specific GWAS. We used IVW as the primary approach followed by sensitivity analyses.

### Ethics

This study used existing publicly available summary statistics reporting genome-wide variant-trait associations for LDL-C and PD. Separate ethical approval was not required.

## Results

### Power calculations

We have adequate power (>76%) to detect modest effects in either direction (0.90 ≥ OR ≥ 1.10) per SD difference in the trait for our MR analyses for LDL-C (Supplementary Table 4). However, we do not have adequate power using only a single genetic variant (e.g., a sentinel variant in *PCSK9*). We thus ran our sex-specific gene-based cis-MR analyses with the four lipid-lowering drug target genes together (*APOB, HMGCR, NPC1L1*, and *PCSK9*), rather than one gene at a time.

### Sex-specific cis-Mendelian randomization analyses

**Figure 1:**
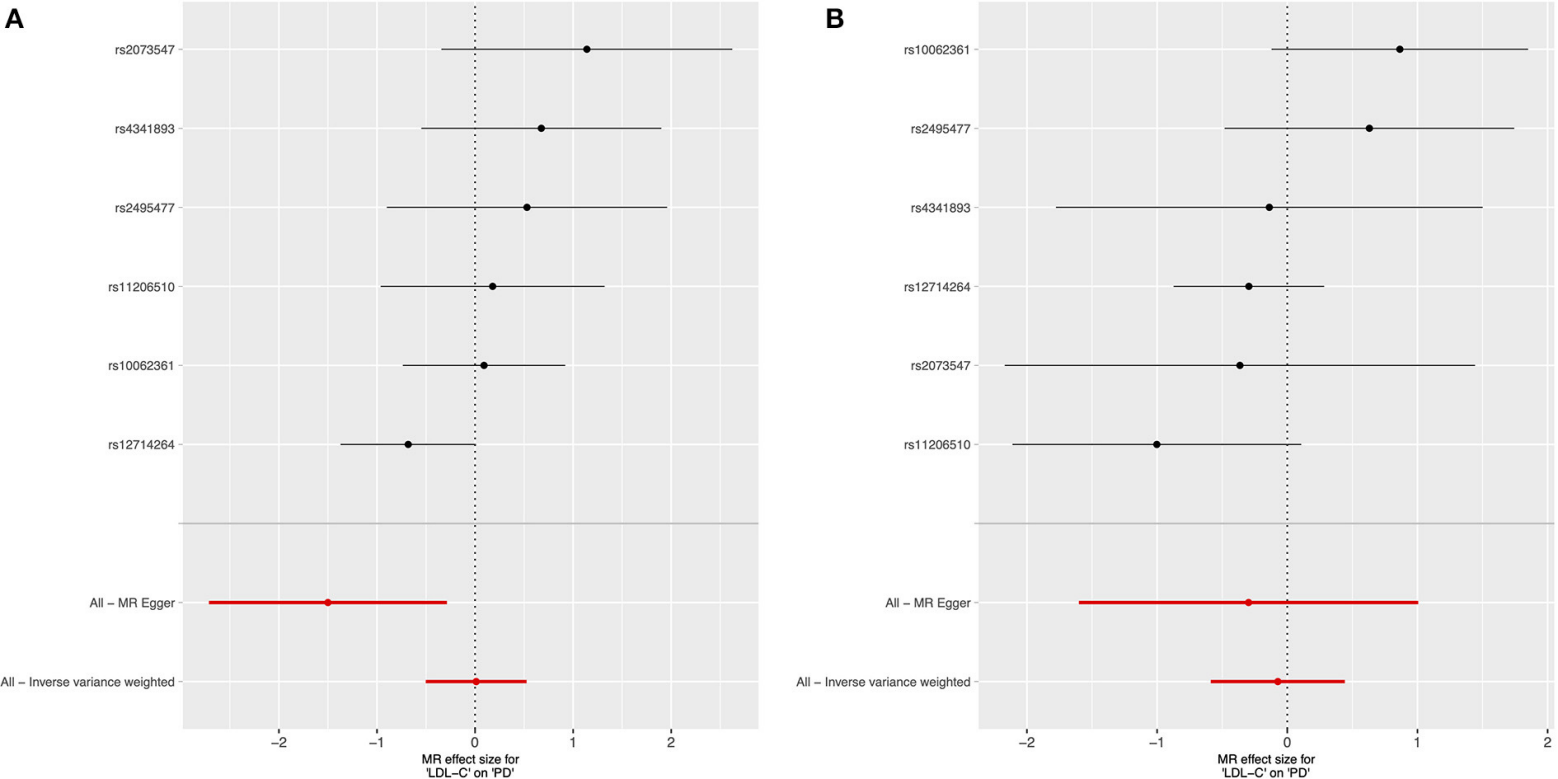
Forest plot of sex-specific cis-Mendelian randomization results for the effect of circulating LDL cholesterol levels on risk of late-onset Parkinson's disease. Female-specific results presented in **(A)**, and male-specific in **(B)**.

We used 6 genetic instruments in our female-specific and male-specific cis-MR analysis (Table 1). We did not find significant evidence for a relationship between LDL-C levels and late-onset PD risk in either the female-specific or male-specific cis-MR analyses (Table 2, Figure 1).

**Table 1:** Genetic instrumental variables for the sex-specific cis-Mendelian randomization analysis between LDL-C gene targets and late-onset PD.

**Genetic instrument for LDL-C**	**Female-specific analysis**				**Male-specific analysis**			
	**LDL-C Beta (SE)**	**LDL-C effect allele**	**PD Beta (SE)**	**PD effect allele**	**LDL-C Beta (SE)**	**LDL-C effect allele**	**PD Beta (SE)**	**PD effect allele**
rs10062361 (*HMGCR*)	0.066717 (0.0039256)	T	0.0061 (0.0282)	T	0.0495 (0.00428)	T	0.0428 (0.0249)	T
rs11206510 (*PCSK9*)	−0.050126 (0.0041043)	C	0.009 (0.0292)	T	−0.0454 (0.00446)	C	−0.0455 (0.0257)	T
rs12714264 (*APOB*)	−0.096566 (0.004815)	T	−0.0658 (0.034)	A	−0.103 (0.00524)	T	−0.0304 (0.0304)	A
rs2073547 (*NPC1L1*)	0.040959 (0.0041382)	G	−0.0467 (0.031)	A	0.0294 (0.00452)	G	0.0107 (0.0271)	A
rs2495477 (*PCSK9*)	−0.037018 (0.0033441)	G	0.0196 (0.027)	A	−0.0428 (0.00366)	G	0.027 (0.0243)	A
rs4341893 (*APOB*)	−0.040996 (0.0033505)	G	0.0277 (0.0256)	A	−0.0275 (0.00365)	G	−0.0038 (0.023)	A

**Table 2:** Sex-specific cis-Mendelian randomization results (estimates for gene targets weighted by LDL cholesterol estimates).

	**Female-specific analysis**	**Male-specific analysis**
	**LDL-C (*****N*** **genetic variants used as instrument = 6)**	
IVW OR (95% CI); *p*	1.01 (0.60–1.69); *p* = 0.96	0.93 (0.55–1.56); *p* = 0.78
Weighted median OR (95% CI); *p*	1.11 (0.62–1.97); *p* = 0.72	0.78 (0.46–1.33); *p* = 0.36
Simple mode OR (95% CI); *p*	1.44 (0.52–4.03); *p* = 0.51	0.75 (0.26–2.22); *p* = 0.63
Weighted mode OR (95% CI); *p*	1.20 (0.49–2.90); *p* = 0.71	0.75 (0.39–1.41); *p* = 0.41
MR-Egger OR (95% CI); *p*	0.22 (0.07–0.75); *p* = 0.015	0.74 (0.20–2.74); *p* = 0.68
MR-Egger intercept (*p*)	0.09 (*p* = 0.009)	0.01 (*p* = 0.73)
${\text{I}}_{\text{GX}}^{2}$ statistic	94.9%	95.8%

We conducted the MR-Egger intercept test to assess whether the intercept significantly differs from 0, which would suggest the existence of directional pleiotropy (that is, the average pleiotropic effect differs from zero) and/or violation of the INstrument Strength Independent of Direct Effect (“InSIDE”) assumption (which states that there is no correlation between the genetic associations with the exposure and the direct effects of the genetic instruments on the outcome). The intercept was not significantly different from 0 in the male-only analysis (*p* = 0.73), but significant for the female-only analysis (*p* = 0.009). As the IVW method requires absence of horizontal pleiotropy to obtain valid causal estimates (91), our results from the MR-Egger intercept test for the female-specific analysis implied that this IVW assumption may be violated and that the IVW results could be biased (moreover the correlation between instrument strength and pleiotropic effects can be large by chance with a small number of instruments) (92). However, we did not find strong evidence for violation of the NO Measurement Error (“NOME”) assumption for the MR-Egger regression estimate, as the ${\text{I}}_{\text{GX}}^{2}$ statistic, a measure of the collective strength of a set of instruments, was >94.9% for both sex-specific analyses (Table 2). Additionally, MR-PRESSO did not identify outlier instruments for either the female-only or male-only analyses (*p* = 0.175 and *p* = 0.221, respectively).

We also assessed the possibility of reverse causality (the possibility of PD influencing LDL-C levels, rather than vice-versa) through Steiger filtering. For both sexes, the instruments explained more variance of the exposure (LDL-C) than the outcome (PD), supporting the direction to be from LDL-C levels influencing PD risk, rather than the opposite. The scatterplots of effect sizes, leave-one out, and funnel plots for the sex-specific cis-MR analyses are presented in Supplementary Figures 2–4, respectively.

### Sex-specific standard Mendelian randomization analyses

We used 28 genetic instruments for the female-specific analysis (independent variants with *p* < 5 × 10^−8^ from the female-only LDL-C GWAS) and 20 for the male-specific analysis (independent variants with *p* < 5 × 10^−8^ from the male-only LDL-C GWAS), which are outlined in Supplementary Table 5. The primary analysis (IVW method) and sensitivity analyses yielded consistent but non-significant estimates. Results are presented in Supplementary Table 6 and visualized in Supplementary Figures 5–8. The MR-Egger intercept test did not suggest evidence of horizontal pleiotropy for the female-only or the male-only analysis (*p* = 0.95 and *p* = 0.53, respectively). MR-PRESSO did not identify outlier genetic instruments for the female-only or the male-only analysis (*p* = 0.225 and *p* = 0.369, respectively).

## Discussion

We did not find significant evidence for a causal effect between genetically-determined LDL-C levels and late-onset PD risk in either the female-only or the male-only MR analyses using genetic variants at lipid drug targets (cis-MR) or genome-wide variants associated with LDL-C levels (standard MR).

In contrast to findings from several observational studies suggesting a negative association between LDL-C and PD risk, our sex-specific MR results, as do most of the current sex-agnostic MR studies, do not provide strong evidence of a causal relationship between LDL-C levels and PD risk (50, 72, 74). Possible explanations for this discrepancy could include confounding factors in observational studies (including reverse causation as well as uncontrolled or imperfectly controlled lifestyle and biological factors in the comparison groups) or limitations of MR analyses (including imperfect validity of the core assumptions and lack of power to detect weaker effects).

The absence of association in our sex-specific framework could suggest that the difference in PD prevalence between the sexes may not be primarily caused by differences in LDL-C levels. There are several mechanisms proposed for the higher risk of PD in men compared to women in addition to that of the effects of lipid metabolism. So far, no evidence has been found of sex-specific common variants involved in PD risk in autosomes (80) or on the X chromosome (93), although genetics studies on this subject with larger sample sizes and taking into account X chromosome inactivation are warranted. However, men and women have innate differences in nigrostriatal dopamine transmission (94–97) and response to stress (98, 99) that can affect their risk for developing PD. Estrogen-modulated differences in mitochondrial function (78) as well as in vulnerability to oxidative stress and neuroinflammation (100) may also account for the relative neuroprotection seen in women for PD development. Furthermore, men may be at greater risk of developing PD due to environmental and lifestyle factors, such as higher rates of traumatic head injuries (77, 101) and increased occupational exposure to agricultural pesticides (77, 102).

In addition to differences between the sexes in PD, there are also well-documented differences in lipid metabolism and profiles between the sexes (with premenopausal women having a more cardioprotective profile than age-matched men due to greater HDL-C and lower LDL-C and triglyceride levels) (103) that result from a complex and complimentary interplay between the actions of sex chromosomes and gonadal hormones. These interact with other factors (such as X chromosome imprinting and inactivation, environmental stimuli, and the gut microbiome) to influence gene expression and protein signaling pathways to produce sexually dimorphic phenotypes (23). Evidence for differences in associations between genetic variants and LDL-C levels through sex-stratified GWAS further supports a genetic contribution to the observed sexual dimorphism in lipid levels (75).

Although we performed several sensitivity analyses, including tests for horizonal pleiotropy, to ensure the robustness and consistency of results, there remain several limitations. First, we note that we are adequately powered to detect modest effects (0.90 ≥ OR ≥ 1.10); it is therefore possible that smaller effects might be missed. Also, it has been established that sample overlap between the exposure and outcome datasets can lead to biased MR estimates. It is unlikely, however, that there is meaningful overlap between our data sources as our exposure data was derived from the UK Biobank and our outcome data was from non-UK Biobank individuals. In addition, recent research has shown that sex-differential participation bias is present in large cohorts, including the UK Biobank, and that such sampling can bias sex-specific estimates in MR analyses (104). This participation bias is a limitation. As for all MR studies, we note that our results cannot be generalized to other populations, such as individuals of different genetic ancestry. Additionally, despite efforts to select valid genetic instruments and datasets derived from individuals of similar genetic ancestry, we cannot be certain that the core assumptions and the assumption of environmental equivalence of the exposure and outcome datasets are met. Furthermore, MR studies assess the relationship between cholesterol levels and PD based on genetic information; hence, the variants are proxies for lifelong LDL-C levels, which may not necessarily reflect LDL-C levels affected by the environment. Finally, here, we defined sex in terms of genetic sex karyotype, specifically the presence of two X chromosomes for females or one X and one Y chromosome for males. Genetic sex is, however, a non-binary spectrum (for instance, individuals can have aneuploidy of the X or Y chromosomes or monosomy of the X), and we recognize our binary classification as a limitation.

Of note, cholesterol has also been linked to the possible pathogenesis of other movement disorders. For example, in Huntington's Disease (HD), an autosomal dominant genetic disorder characterized by the expansion of the CAG repeats in the gene encoding for the huntingtin protein (105), important perturbations in both central nervous system (CNS) and whole-body cholesterol metabolism have been described. In the CNS, the mutated huntingtin protein induces downregulation of cholesterol biosynthesis (106–108) and reduces cholesterol's cellular efflux to ApoE, negatively impacting its transportation from astrocytes to neurons (109, 110). In human post-mortem tissues, increased cholesterol was also found in the caudate (111) and putamen (112). The alteration in cholesterol metabolism is theorized to contribute to HD pathogenesis by various neuronal processes, including enhanced vulnerability to excitotoxicity, increased neuroinflammation, and impaired synaptic transmission (113). There is also evidence of a decrease in plasma 24S-OHC levels (theorized to be caused by neurodegeneration) (114) as well as in peripheral cholesterol biosynthesis and elimination (115), where the huntingtin protein is also ubiquitously expressed (116). Currently, while the picture is incomplete, correction of CNS cholesterol metabolism is being investigated as a possible therapeutic avenue for HD, with promising results in animal studies (117–120). So far, to our knowledge, there are no MR studies on the association between cholesterol and other movement disorders.

In conclusion, our sex-specific MR analyses do not provide evidence of a causal relationship between genetically-determined LDL-C levels on PD risk, which agrees with findings from the sex-agnostic analyses conducted by Williams et al. (50). Nevertheless, sex differences in lipid metabolism and profiles, in the genetic basis of LDL-C levels, in the prevalence and manifestation of PD, and in other factors are known to exist. These differences highlight the importance of future investigation of sex-specific factors. From the genetics perspective, with individual-level data, testing LDL-C sex-specific polygenic scores for association with PD case-control status could provide another approach to assess whether genetically-determined LDL-C is associated with PD risk. Going forward, integration of research from multiple fields is necessary to clarify the biological mechanisms by which cholesterol levels influence PD risk and to better interpret results from both observational and MR studies. The latter have already shown great promise as an important methodological component of this multi-disciplinary approach. As sample sizes in genetic studies continue to increase, permitting better powered sex-stratified efforts, we hope that sex-aware analyses become mainstream and yield important discoveries.

## Data availability statement

Publicly available datasets were analyzed in this study. This data can be found here: the sex-stratified UK Biobank LDL cholesterol (rank inverse normal transformed) summary statistics analyzed in this study are available for download through the Round 2 of the Neale lab UK Biobank GWAS analyses released August 1, 2018 (http://www.nealelab.is/uk-biobank/), specifically: 30780_irnt.gwas.imputed_v3.female.varorder.tsv.bgz and 30780_irnt.gwas.imputed_v3.male.varorder.tsv.bgz. The sex-stratified Parkinson's disease summary statistics analyzed in this study are from Blauwendraat et al. (80) and are available on the International Parkinson Disease Genetics Consortium Resources page (https://pdgenetics.org/resources). All R code for running the MR analyses and constructing the plots are available in a GitHub repository (https://github.com/GaglianoTaliun-Lab/ldl_pd_mr).

## Ethics statement

Ethical review and approval was not required for the study on human participants in accordance with the local legislation and institutional requirements. Written informed consent for participation was not required for this study in accordance with the national legislation and the institutional requirements.

## Author contributions

YZ and SG drafted the manuscript, performed analyses, and contributed to design and/or direction. SG conceived the project. All authors approved the final manuscript.

## Funding

SG was funded by a Junior 1 award from the Fonds de recherche du Québec - Santé (FRQS; https://frq.gouv.qc.ca) and by Operational Funds from the Institut de valorisation des données (IVADO; https://ivado.ca).

## Conflict of interest

The authors declare that the research was conducted in the absence of any commercial or financial relationships that could be construed as a potential conflict of interest.

## Publisher's note

All claims expressed in this article are solely those of the authors and do not necessarily represent those of their affiliated organizations, or those of the publisher, the editors and the reviewers. Any product that may be evaluated in this article, or claim that may be made by its manufacturer, is not guaranteed or endorsed by the publisher.
