# Supplementary material for: Lipid-lowering drug targets and Parkinson's disease: A sex-specific Mendelian randomization study
Source: Front Neurol. 2022 Sep 1;13:940118. doi: 10.3389/fneur.2022.940118 (PMC9477004; doi:10.3389/fneur.2022.940118)
Supplement: Supplementary file 1 [file Data_Sheet_1.docx]

Supplementary Material

[SUPPLEMENTARY TEXT 2](#_Toc297314522)

[SUPPLEMENTARY FIGURES 4](#_Toc231711945)

[SUPPLEMENTARY TABLES 12](#_Toc980739118)

**Supplementary Text**

Literature review of the possible role of cholesterol levels on Parkinson’s disease risk: additional sex-agnostic Mendelian randomization studies

Benn et al. ^1^, for instance, used both an observational and MR (allele score-based) design as part of their cohort study (460 PD cases and total sample of 111,194 Danish subjects). In their observational study, they identified a negative association between circulating LDL-C and PD risk for lower categories of LDL-C levels (comparing participants with LDL-C <1.8 mmol/L vs. those with ≥4.0 mmol/L), but the association disappeared when examining continuous LDL-C levels. Genetic analyses using an allele score constructed from five variants affecting LDL-C metabolism (from *PCSK9* and *HMGCR* genes) found no significant association between LDL-C and PD risk (adjusted RR for 1mmol/L lower LDL-C was 1.02 [0.26, 4.00]). The authors hypothesized that the association found in lower categories of LDL-C in the observational design may be due to reverse causation and thus concluded that lower LDL-C did not significantly increase PD risk.

On the other hand, Fang et al. ^2^ reported a negative association between both LDL-C or TC and PD risk in a nested case-control design (3769 PD cases and total sample size of 610,141). The negative association was supported by a two-sample MR analysis using public PD GWAS data and PD cohorts from 23andMe (38,426 PD cases and 807,269 controls of European ancestry), and 244 genetic variants to proxy for TC levels and 204 variants for LDL-C levels (LDL-C analysis OR 0.96 [0.92, 0.99], p= 0.02 and TC analysis OR 0.94 [0.90, 0.98], p= 0.002). Fang et al. concluded that these results offer evidence of a possible protective effect of cholesterol levels on PD risk.

A genetic risk score-based MR cohort study by Liu et al. in 2021 ^3^ also did not find evidence of LDL-C having a causal effect on PD risk. The authors of the study examined the BioVu cohort (553 PD cases and 40,640 controls) and, for replication, the eMerge cohort for (538 cases and 19,330 controls) using a weighted genetic risk score for the *HMGCR* gene considering six independent LDL-C associated variants. The risk score was not significantly associated with PD risk in either the BioVu cohort (OR 1.30 [1.07, 1.58], p= 0.007, as it did not meet their Bonferonni correction of <0.002) or in the eMerge cohort (with 5/6 variants available, OR 0.93 [0.75, 1.16], p= 0.53). Results in both cohorts were similar after adjusting for statin use.

Methods for the literature review of the possible role of cholesterol levels on Parkinson’s disease risk

Observational epidemiological studies were identified mainly from PubMed searches as well as from reference lists of meta-analyses and systematic reviews. First, PubMed was searched using the MeSH terms (("Cholesterol"[Mesh]) AND "Parkinson Disease"[Mesh]) AND "epidemiology" [Subheading]. We also searched for systematic reviews and meta-analyses by entering the term “Cholesterol Parkinson’s Disease” in the PubMed search bar and restricting article types to Meta-Analysis and Systematic Review. Of the three articles identified in the last step, the studies analysed by the two most recent meta-analyses/systematic reviews (Fu et al. ^4^ and Jiang et al. ^5^) were considered. Finally, the correspondence between Huang et al. and Scigliano et al. ^6, 7^ was found through Google searches of their articles. We included the primary research articles referred to in their correspondence as a starting point in our search for additional literature.

The articles met the following inclusion criteria:

1. Case-control or cohort studies that examined the association between serum TC and/or circulating LDL-C levels with PD risk
2. Peer-reviewed and published
3. Published in the English Language
4. Published after January 1, 2005 and before December 1, 2021
5. Studies with more than 100 PD cases
6. Studies that were mentioned in the correspondence between Scigliano et al. ^6^ and Huang et al. ^7^, regardless of case count

The Mendelian randomization studies were identified through PubMed by entering “Cholesterol Parkinson’s Disease Mendelian randomization” in the search bar. The same search was carried out in Google Scholar, and the first three pages of results were examined for relevant additional studies (none were found).

The articles met the following inclusion criteria:

1. One-sample or two-sample Mendelian randomization studies that examined the association between serum TC and/or circulating LDL-C with PD risk
2. Peer-reviewed and published
3. Published in the English language
4. Published after January 1, 2005 and before December 1, 2021
5. Studies with more than 450 PD cases (only for studies based solely on first-degree relatives with PD)

**Supplementary Figures**

**
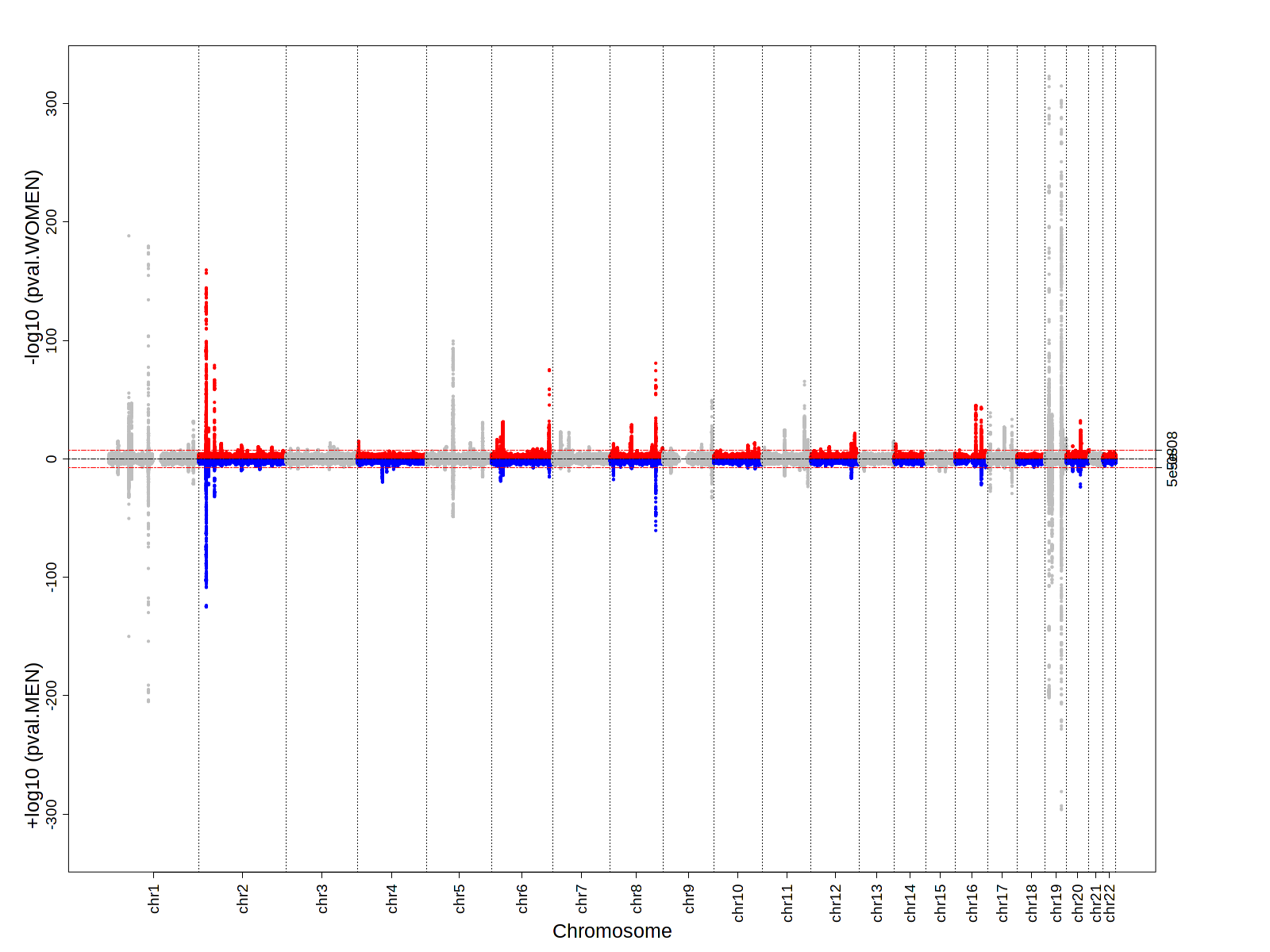
**

**a**

**
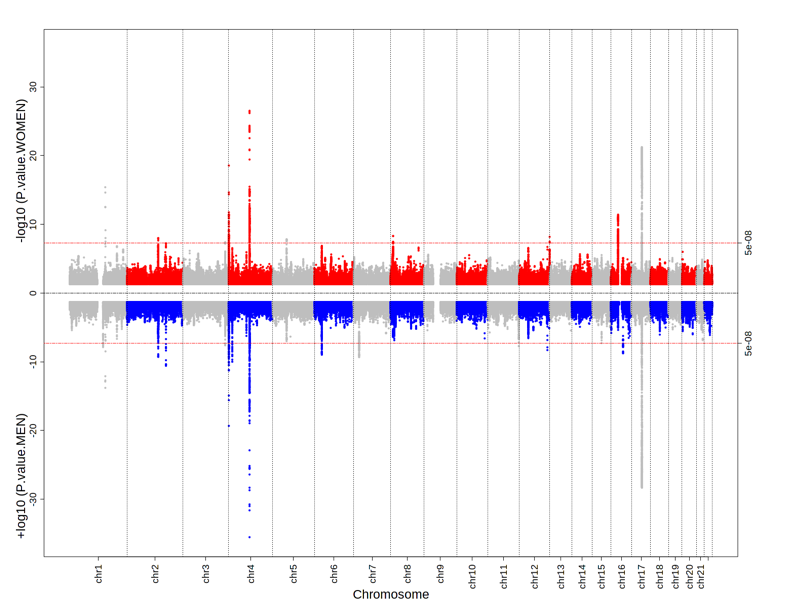
**

**b**

**Supplementary Figure 1. Miami plots of sex-stratified genome-wide association results** from which the variant-exposure and variant-outcome association summary statistics were obtained. Directly measured LDL-C (exposure) results presented in **a**, and late-onset Parkinson’s disease (outcome) in **b**. In each plot, the dashed red line denotes the genome-wide significance threshold of p=5x10^-8^. In each plot, the female-only association results are presented in the top half and the male-only results are in the bottom half.

**a**

**
 b**

**Supplementary Figure 2. Scatterplot of sex-specific cis-Mendelian randomization results** for the effect of LDL-C levels on risk of late-onset Parkinson’s disease. Female-specific results presented in **a**, and male-specific in **b**.

**a**

 **b**

**Supplementary Figure 3. Leave-one-out analysis of sex-specific cis-Mendelian randomization results** for the effect of LDL-C levels on risk of late-onset Parkinson’s disease using the inverse variance weighted approach. Female-specific results presented in **a**, and male-specific in **b**.

**a**

**b**

**Supplementary Figure 4. Funnel plot of sex-specific cis-Mendelian randomization results** for the effect of LDL-C levels on risk of late-onset Parkinson’s disease. Female-specific results presented in **a**, and male-specific in **b**.

**a**

**b**

**Supplementary Figure 5. Forest plot of sex-specific genome-wide (standard) Mendelian randomization results** for the effect of LDL-C levels on risk of late-onset Parkinson’s disease. Female-specific results presented in **a**, and male-specific in **b**.

**a**

**b**

**Supplementary Figure 6. Leave-one-out analysis of sex-specific standard Mendelian randomization results** for the effect of LDL-C levels on risk of late-onset Parkinson’s disease using the inverse variance weighted approach. Female-specific results presented in **a**, and male-specific in **b**.

**a**

**b**

**Supplementary Figure 7. Funnel plot of sex-specific standard Mendelian randomization results** for the effect of LDL-C levels on risk of late-onset Parkinson’s disease. Female-specific results presented in **a**, and male-specific in **b**.

**a**

**b**

**Supplementary Figure 8. Scatterplot of sex-specific standard Mendelian randomization results** for the effect of LDL-C levels on risk of late-onset Parkinson’s disease. Female-specific results presented in **a**, and male-specific in **b**.

**Supplementary Tables**

**Supplementary Table 1. STROBE-MR ^8, 9^ checklist of recommended items to address in reports of Mendelian randomization studies**

| **Item** | **Complete/location** |
| --- | --- |
| 1. **Title and Abstract:** Indicate Mendelian randomization (MR) as the study’s design in the title and/or the abstract if that is a main purpose of the study. | Done |
| **Introduction** |  |
| 1. **Background:** Explain the scientific background and rationale for the reported study. Is causality between exposure and outcome plausible? Justify why MR is a helpful method to address the study question. | In the introduction, we discuss why circulating LDL levels is a plausible causal exposure for late-onset PD, and there could be sex-specific effects. We describe various mechanisms for either a positive or an inverse relationship between LDL levels and Parkinson’s disease risk. We also discuss why Mendelian randomization is a reasonable approach to investigate a potential relationship between LDL-C and PD in a sex-specific manner. |
| 1. **Objectives:** State specific objectives clearly, including pre-specified causal hypotheses (if any). State that MR is a method that, under specific assumptions, intends to estimate causal effects. | In the introduction we describe the objectives of our study. We state that under specific assumptions, MR intends to estimate causal effects. |
| **Methods** |  |
| 1. **Study design and data sources:** Present key elements of study design early in the paper. Consider including a table listing sources of data for all phases of the study. For each data source contributing to the analysis, describe the following:   a) Setting: Describe the study design and the underlying population, if possible.  Describe the setting, locations, and relevant dates, including periods of recruitment,  exposure, follow-up, and data collection, when available  b) Participants: Give the eligibility criteria, and the sources and methods of selection of participants. Report the sample size, and whether any power or sample size calculations were carried out prior to the main analysis  c) Describe measurement, quality and selection of genetic variants  d) For each exposure, outcome and other relevant variables, describe methods of assessment and diagnostic criteria for diseases  e) Provide details of ethics committee approval and participant informed consent, if relevant | a-b) Available information about the GWAS studies, including sample size, is provided in the description of the datasets, and characteristics of the studies included in the Parkinson’s disease meta-analysis are listed in a Supplementary Table. Further information is given in each of the original GWAS publications.    c) Selection of genetic variants is described. Power calculations for the MR analyses are reported.  d) For Parkinson’s disease, the diagnosis criteria are provided in the description of the dataset.    e) As this study used only publicly available summary statistics, ethics approval was not required. |
| 1. **Assumptions:** Explicitly state the three core IV assumptions for the main analysis (relevance, independence, and exclusion restriction) as well assumptions for any additional or sensitivity analysis | We explicitly state the three core IV assumptions. We also explain additional assumptions for IVW and MR-Egger. |
| 1. **Statistical methods main analysis:**   Describe statistical methods and statistics used  a) Describe how quantitative variables were handled in the analyses (i.e., scale, units, model)  b) Describe how genetic variants were handled in the analyses and, if applicable, how their weights were selected  c) Describe the MR estimator (e.g. two-stage least squares, Wald ratio) and related statistics.  Detail the included covariates and, in case of two-sample MR, whether the same covariate set was used for adjustment in the two samples  d) Explain how missing data were addressed  e) If applicable, indicate how multiple testing was addressed | a) Within the description of the datasets, we describe the scale and units for LDL in the UK Biobank cohort, and how the variable was transformed for the association analysis  b) We describe how genetic variants were handled in the part describing the selection of genetic instruments.  c) We conduct two-sample MR. We state the covariates used in the GWAS analyses in the description of the datasets.  d) For genetic variants absent from one of the two GWAS datasets, we describe how a corresponding proxy was chosen.  e) Not applicable. |
| 1. **Assessment of assumptions:** Describe any methods used to assess the assumptions or justify their validity | We assess the validity using sensitivity analyses, including MR-Egger regression. We only select instruments with genome-wide significant statistical association (p<5x10^-8^) with the exposure from large GWAS.  We verify that the selected instruments have non-significant p-values in the outcome data. |
| 1. **Sensitivity analyses:** Describe any sensitivity analyses or additional analyses performed (e.g. comparison of effect estimates from different approaches, independent replication, vias analytic techniques, validation of instruments, simulations) | We run MR-Egger, weighted median, simple mode and weighted mode as sensitivity analyses to complement the IVW with random effects primary sex-specific cis-MR analyses. To test for possible outliers and for horizontal pleiotropy, we run MR-PRESSO and the MR-Egger intercept test, respectively. We also present results from complimentary standard (genome-wide) sex-specific MR to compliment the cis-MR analyses. |
| 1. **Software and pre-registration:**   a) Name statistical software and package(s), including version and settings used  b) State whether the study protocol and details were pre-registered (as well as when and  where) | a) All statistical software and settings used are provided.  b) The study protocol and details were not pre-registered, which is stated in the methods. |
| **Results** |  |
| 1. **Descriptive data**   a) Report the numbers of individuals at each stage of included studies and reasons for exclusion. Consider use of a flow-diagram  b) Report summary statistics for phenotypic exposure(s), outcome(s) and other relevant variables (e.g. means, SDs, proportions)  c) If the data sources include meta-analyses of previous studies, provide the assessments of heterogeneity across these studies  d) For two-sample MR:  i. Provide justification of the similarity of the genetic variant-exposure associations between the exposure and outcome samples  ii. Provide information the number of individuals who overlap between the exposure and outcome data sources | a) Information on the study participants is provided in the description of the datasets.  b) We report the summary statistics for our instruments in Table 1 (se-specific cis-MR analyses) and Supplementary Table 3 (sex-specific standard MR analyses), as well as in a GitHub repository. Full summary statistics are also available from each GWAS as described in the description of the datasets.  c) We give information available on the various studies meta-analyzed for the PD summary statistics used Supplementary Table 1.  d) The exposure (LDL) association results are derived from analyses conducted in the UK Biobank participants. We describe that for our two-sample MR setting, we use Parkinson’s disease (outcome) summary statistics that have excluded UK Biobank samples to ensure as low as possible overlap between exposure and outcome data sources. We describe also how both samples are composed of adult individuals with European-ancestry. |
| 1. **Main results:**     a) Report the associations between genetic variant and exposure, and between genetic variant and outcome, preferably on an interpretable scale  b) Report MR estimates of the relationship between genetic variant and exposure, and between genetic variant and outcome, and the measures of uncertainty from the MR analysis, on an interpretable scale, such as odds ratio, or relative  risk, per SD difference  c) If relevant, consider translating estimates of relative risk into absolute risk for a meaningful time-period  d) Consider any plots to visualize results (e.g. forest plot, scatterplot of associations between genetic variants and outcome versus between genetic variants and exposure) | Results are given in terms of odds ratio and 95% confidence intervals. We visualize results for the female-specific and male-specific analyses using a forest plot in Figure, and as supplementary figures: a scatter plot of genetic variant effects on exposures versus outcomes, a funnel plot and a leave-one-out analysis plot. |
| 1. **Assessment of assumptions:**     a) Report the assessment of the validity of the assumptions  b) Report any additional statistics (e.g., assessments of heterogeneity across genetic variants, such as I^2^, Q statistic or E-value) | a) We assess the validity using sensitivity analyses, including MR-Egger regression. We only select instruments with genome-wide significant statistical association (p<5x10^-8^) with the exposure from large GWAS.  b) We present the I^2^_GX_ value for the sex-specific MR analyses. |
| 1. **Sensitivity and additional analyses:**   a) Report any sensitivity analyses to assess the robustness of the main results to violations of the assumptions  b) Report results from other sensitivity analyses or additional analyses  c) Report any assessment of direction of causality (e.g., bidirectional MR)  d) When relevant, report and compare with estimates from non-MR analyses  e) Consider any additional plots to visualize results (e.g., leave-one-out analyses) | a) We present results from MR-Egger, weighted median, simple mode and weighted mode as sensitivity analyses to complement the IVW with random effects primary sex-specific cis-MR analyses. To test for possible outliers and for horizontal pleiotropy, we run MR-PRESSO and the MR-Egger intercept test, respectively.  b) We also present results from complimentary standard (genome-wide) sex-specific MR to compliment the cis-MR analyses. For the standard MR, we also present IVW with random effects as the primary MR approach as well as the following sensitivity analyses: MR-Egger, weighted median, simple mode and weighted mode. To test for possible outliers and for horizontal pleiotropy, we run MR-PRESSO and the MR-Egger intercept test, respectively.  c) We report Steiger filtering results for the cis-MR analyses.  d) We review the pertinent estimates present in the literature from observational studies for the possible effect of LDL levels on Parkinson’s disease risk.  e) We show leave-one-out analysis plots in the Supplement. |
| **Discussion** |  |
| 1. **Key results:** Summarize key results with reference to study objectives | We describe key results in the first paragraph of the discussion section. |
| 1. **Limitations:**   Discuss limitations of the study, taking into account the validity of the IV assumptions, other sources of potential bias, and imprecision. Discuss both direction and magnitude of any potential bias, and any efforts to address them | Limitations are discussed in the discussion, including the potential participation bias in the UK biobank dataset as well as the possibility that the core IV assumptions were not met despite our best efforts. |
| 1. **Interpretations:**   a) Meaning: Give a cautious overall interpretation of results in the context of their limitations in comparison with results from other relevant studies  b) Mechanism: Discuss underlying biological mechanisms that could drive a potential causal relationship between the investigated exposure and the outcome, and whether the gene-environment equivalence assumption is reasonable. Use causal language carefully, clarifying that IV estimates may provide causal effects only under certain assumptions  c) Clinical relevance: Discuss whether the results have clinical or public policy relevance, and to what extent they can inform effect sizes of possible interventions | a) We present a cautious overall interpretation of results.  b) We discuss the possible role of LDL levels on Parkinson’s disease risk.  c) We discuss the importance of sex-specific Mendelian randomization analyses to further personalized medicine. |
| 1. **Generalizability:** Discuss the generalizability of the study results (a) to other populations, (b) across other exposure periods/timings, and (c) across other levels of exposure | We present limitations and potential caveats in the discussion, including generalizing our findings to other populations. |
| 1. **Funding:** Describe sources of funding and the role of funders in the present study, and if applicable, sources of funding for the databases and original study or studies on which the present study is based | We have reported all sources of funding. |
| 1. **Data and data sharing:** Provide the data used to perform all analyses or report where and how the data can be accessed, and reference these sources in the article. Provide the statistical code needed to reproduce the results in the article, or report whether the code is publicly available and if so, where | We give access information to all data used in the study and list which software were used. We also provide code and input files to run the analyses and construct the plots on a GitHub repository. |
| 1. **Conflicts of Interest:** All authors should declare all potential conflicts of interest | All authors declared conflicts of interest (none reported). |

**Supplementary Table 2. Overview of sex differences for cholesterol levels and Parkinson’s Disease**

|  | PD | | Cholesterol | |
| --- | --- | --- | --- | --- |
|  | Men | Women | Men | Women |
| Genetics | Autosomes: no significant genetic difference between PD cases of either sex   - High genetic correlation between sexes - Similar heritability between sexes - No difference in cumulative genetic risk score between male and female PD cases ^10^   X-chromosome-wide association study (XWAS): no significant sex-specific results   - 2 significant loci on chr X, but these have similar effects in both sexes ^11^ - Studies incorporating X inactivation and larger sample sizes are warranted   Avenue of investigation: evidence of regulation of nigrostriatal DA system by SRY gene ^12^ | | Sex-stratified GWAS show evidence of heterogeneity by sex for TG, LDL-C, HDL-C^13^  Sex-chromosome complement influences lipid profile:   - Evidence for protective effects of X sex chromosome complement on cholesterol levels: - Turner Syndrome (XO): higher TG and LDL-C levels than age-matched XX women with premature ovarian failure ^14^ - Klinefelter Syndrome (XXY): before puberty have increased body fat mass ^15^ – in adulthood, have higher LDL-C and TG levels, lower HDL-C levels, greater fat mass, and higher risk of metabolic syndrome compared to age-matched XY men^16^ - Four Core Genotype mice model: XX or XXY genotype show increased adiposity compared to XY or XO genotype, regardless of gonadal sex (gonadally male or female) ^17^ - Y chromosome variation may influence lipoprotein profiles, e.g. LDL-C, TC, and HDL-C ^18^ ^18^   Both gonadal hormones and sex chromosomes influence gene expression   - Possible mechanisms for effects of X chromosome complement: parental X chromosome imprinting, genes that escape X inactivation ^19^ - GTEx in humans show that thousands of genes are differentially expressed in female vs male tissues ^20^ - Mice: sex-specific genetic expression in adipose tissue, liver, skeletal muscle, and brain ^21^ - Mice: sex-specific gene expression modules in adipose tissue and liver correlated with body fat and lipid levels – many modules regulated by gonadal hormones ^22^ - Mice: sex chromosomes affect expression of autosomal genes ^23^ | |
| Metabolism | Sex differences in nigrostriatal dopamine (DA) transmission, e.g:   - in striatal DAergic neuron dynamics ^24-27^ - in the “functional relationship between regional DA release and motor performance, affect, and cognitive function” ^26^     Explanations for relative neuroprotection in women:   - Up-regulated in women: genes for neuronal maturation and signal transduction - Up-regulated in men: genes that, when mutated, are involved in PD pathogenesis ^28^ - Evidence that estrogens are responsible for relative neuroprotection in women: 17β-estradiol (E2) has neuroprotective effects^29^ - Female DAergic cells generally less vulnerable to degeneration than male DAergic cells ^29^   Sex differences in neuroinflammatory processes (involving microglia and astrocytes):  *Astrocytes*:   - sexually differentiated in morphology; sensitive to hormones ^30, 31^ - Circulating estrogens have anti-inflammatory effects on astrocytes ^32^ - In response to injury, astrocytes synthesize estradiol and up-regulate estrogen receptors ^33^ - Toxin-induced PD model in mice: male astrocytes have greater, more deleterious inflammatory response, while female astrocytes promote survival ^34^   *Microglia*:   - In mice: transcriptome of microglia is sexually differentiated, possibly by neonatal estrogen priming that induces permanent phenotypes with different reactivities to environmental and hormonal stimuli (male microglia have greater capacity to react to inflammatory stimuli)^35^ - Microglial transcriptome is more developmentally mature in men than in women^36^ - Gonadal hormones also have regulatory effects on microglia^37^ - Toxin-induced PD mice models: estradiol treatment suppressed microglia inflammatory activation and striatal DA loss ^38^   *Oxidative stress*:   - Strong sexual dimorphism in mitochondrial function^29^ - In PD patients: sex differences in prooxidant-antioxidante balance and malondialdehyde levels in plasma^39^ - In rats: generally, female mitochondria in the brain have less oxidative stress and damage, but have lower calcium uptake ^40-42^ - In mice: ER agonists stimulate mitochondrial functions and reduces oxidative stress^43^ - Female brain: higher normalized neuromelanin-rich volume – neuromelanin scavenges toxic substances (ex: iron) ^44^ - Estrogens regulate iron metabolism – striatum of male mice more susceptible to iron accumulation than female mice^45^ and, at equivalent plasma levels of iron, men are at higher risk of PD than women^46^   Further insights from animal studies:   - Toxin-induced models of PD: female rats show resistance to nigrostriatal dopaminergic lesions up to a certain threshold ^12, 47, 48^ - Toxin-induced models of PD: estradiol (endogenous or exogenous) protects against striatal dopamine loss in female rats up to a certain threshold^12, 47^ - Toxin-induced models of PD: testosterone and estradiol might worsen striatal dopamine loss in male rats up to a certain threshold ^12, 49^ - Sex hormones do not affect survival of dopaminergic cells in substantia nigra –protective effects of estradiol in female rodents may be from compensatory mechanisms in surviving dopaminergic neurons (neuroprotection)^12, 48, 50^ - SNc DAergic cell number depends on expression of SRY gene in male rats – silencing decreases number of neurons and induces motor deficits ^51^ - Sex-specific responses to stress might predispose to toxin-induced PD in rats^12^   Environmental factors that may increase risk of PD:   - Greater occupational exposure to pesticides (ex: paraquat) in men^12, 52^ - More traumatic brain injuries in men^12, 53^ - Lower activity levels, especially in older women^54^ - Sexually dimorphic stress response ^55, 56^ | | Testosterone   - Signals through androgen receptor ^57^, other non-genomic signaling pathways, and regulation of intracellular calcium^58^ - Androgen receptors play more important role in visceral fat tissue^59^   Testosterone facilitates central adipose storage^60^   - Androgen deficiency in men may lead to accumulated visceral fat, production of inflammatory adipokines, and predisposition to metabolic syndrome ^59^   *Androgens’ effects on cholesterol metabolism*   - Little is known about the effects of androgens on cholesterol metabolism. Postulated to also affect lipid levels through actions in liver, adipose tissues, and CNS^61^ - Little evidence that they are important determinants of LDL-C^62^   Effects of androgens on leptin signalling in hypothalamus is unclear^63^: importance of androgen signalling might be secondary to its role as substrate in estrogen synthesis   - In rats: greater aromatase activity in satiety centres in males rats than females^64^   *Administration of exogenous steroid hormones partially recaptures clinical differences seen between premenopausal women and men:*   - Testosterone and its derivatives (aromatizable and non aromatizable) administered in men and women decreases HDL-C (aromatization reduces decrease^65^)^66^   Testosterone administered in:   - Hypogonadal men as replacement doses: <5% decrease in LDL-C^66, 67^ - In eugonadal men^65^ and female-to-male transsexuals^68, 69^: no effect on LDL-C - In peri- and postmenopausal women: may increase LDL-C when given orally (but not when given transdermally)^70^ | Estrogen and progesterone   - Estrogens act through 3 receptors: ERα, ERβ, and GPER^61^ - In mice: deletion of GPER increases LDL-C^71^ - Estrogen receptors play more important role in subcutaneous fat tissue^59^   In premenopausal women, estrogens and progestins   - Protect against abdominal obesity and insulin resistance - Promote fat storage in gluteal-femoral (subcutaneous) fat pads^72^   *Female hormones’ effects on cholesterol metabolism*   - Estrogens play role in regulation of plasma lipid levels by actions in liver (major tissue that impacts lipid metabolism in response to estrogen signaling^61^), adipose tissues, and CNS^61^   At comparable adiposity, premenopausal women have higher leptin than men^73^:   - Leptin: antiobesity effects^74^; synthesized in subcutaneous fat, estrogen regulates ^75, 76^ - Leptin and estrogen on hypothalamus may increase sympathetic nervous system activity and prevents increase of visceral adipocyte pool in premenopausal women^74^   *Administration of exogenous steroid hormones partially recaptures clinical differences seen between premenopausal women and men:*   - Transdermal 17β-estradiol administered in men^77^ and women (pre-^78^ and postmenopausal^79^) influences HDL-C, LDL-C and TG levels - Exogenous progestogens: - Reduce plasma TG - Decrease HDL-C - Little effect plasma LDL-C^66, 79-81^   *Other possible sources of sex dimorphism in lipid metabolism:*   - Sex-specific effects for GI absorption and intracellular trafficking of free fatty acids ^82^,^63^ - higher β-oxidation protein abundance in skeletal muscle in women than in men^83^ - Sex-specific activity of transcription factors regulating lipid metabolism (e.g. SREP, PPAR family, LXR family, FXR)^63^ |
| Clinical Presentation  (age of onset,  manifestations) | - Sexual dimorphism in prodromal PD^84^ - Greater incidence rate: around 1.5 times that of women^85^ - Earlier age at onset^86^ - Low body mass associated with reduced survival time^87^   Motor symptoms:   - Later development of freezing of gait^88^ - Higher risk for camptocormia^89^ - More severe rigidity^90^   More prevalent symptoms:   - Rapid eye movement sleep behaviour disorder^91, 92^ - Daytime sleepiness - Dribbling - Sexual dysfunction^93^   Cognitive skills:   - Worse recognition of facial emotions^94^ - Worse cognitive abilities except for visuospatial abilities^95-97^ - Greater impulse control disorders^98, 99^ - Health-related quality of life: associated with more negative scores in cognition domain^100^ - Lower satisfaction in second half of life^101^ | - Sexual dimorphism in prodromal PD^84^ - Higher mortality rate and faster disease progression^102^ - PD-associated dementia has greater impact on life expectancy^103^   Motor symptoms  tend to emerge later^90^   - Tremor: more common as first symptom^86^ - Higher risk for postural instability and falling^104^   More prevalent symptoms:   - Nervousness - Fatigue - Sadness - Depression - Constipation - Restless legs - Pain - Loss taste or smell - Weight changes - Excessive sweating^93^ - Critical dysphagia^105^   Cognitive skills:   - Worse visuospatial cognition - Better symbol digit modalities test - Better verbal fluency - Better overall cognition^97^ - Health-related quality of life: associated with more negative scores in physical-functioning and socioemotional domains^100^ | - Higher % lean mass - More visceral adipose tissue^62, 106^   More atherogenic, less cardioprotective lipid profile than age-matched premenopausal women^66, 107^ | - Higher % fat mass - More subcutaneous adipose tissue^62, 106^   Premenopausal women: less proatherogenic, more cardioprotective lipid profile than age-matched men:   - Greater HDL-C - Lower LDL-C, VLDL-C, total TG, and VLDL-TG^66, 107^ - LDL: smaller concentration but larger average size^66, 108^ - HDL: similar concentration but larger average size^66, 109^   Premenopausal women protected from myocardial infarction compared to age-matched men   - Onset of first MI around 10 years later than men - Any age: 1/3-1/2 risk of CV disease vs. men^61^   But, have higher incidence of ischemic stroke^110^  With menopause:   - increase in TC - increase in LDL-C - No change in HDL-C^111^ - vs men: similar LDL-C but higher HDL-C^66^ - However, still have lower risk of CV disease than men^61, 112^ - Fat distribution: increase in intra-abdominal fat^113^ |
| Drug response | Evidence of male-female differences in efficacy, tolerability and pharmacokinetics for PD medications (e.g. levodopa), but not yet well-studied^114^   - Women have greater levodopa bioavailability and lower clearance levels^115, 116^ - Greater risk for motor complications associated with levodopa^104, 117^, but this might be because of greater dosage per kilogram body weight^118^ - Administration of estrogen in postmenopausal women might improve symptoms and levodopa-associated dyskinesia^119, 120^ | | Cholesterol-lowering approaches with statins may be equally effective in men and women, however:   - Trials enroll fewer women than men^121^ - Compared to men, women are more likely to decline statin therapy, and more likely to discontinue therapy because of side effects^122^ - Women are more likely than men to have statin-induced myopathy ^123^ | |

CV= cardiovascular; GWAS= genome-wide association study; HDL-C= high density lipoprotein cholesterol; LDL-C= low density lipoprotein cholesterol; PD= Parkinson’s disease; SNc DAergic = substantia nigra pars compacta dopaminergic; TC= total cholesterol; TG= triglycerides; VLDL-C= very low density lipoprotein cholesterol

**Supplementary Table 3. Sex-stratified case-control counts for cohorts included in the Parkinson’s disease meta-analysis used as the outcome in the Mendelian Randomization analyses**

| **Cohort name** | **N male cases** | **N male controls** | **N female cases** | **N female controls** |
| --- | --- | --- | --- | --- |
| Dutch GWAS (PMID:21248740) | 486 | 877 | 279 | 1105 |
| Finnish Parkinson's | 209 | 104 | 177 | 388 |
| German GWAS (PMID:19915575) | 436 | 490 | 276 | 452 |
| Harvard Biomarker Study (HBS) | 346 | 181 | 181 | 291 |
| McGill Parkinson's | 379 | 467 | 201 | 438 |
| Myers-Faroud (PMID:22451204) | 439 | 307 | 311 | 470 |
| NeuroX - dbGaP (phs000918.v1.p1) | 3491 | 3232 | 1914 | 2572 |
| NIA PD GWAS (PMID:19915575) | 500 | 1420 | 341 | 1581 |
| Oslo Parkinson's Disease Study | 306 | 267 | 170 | 195 |
| Parkinson's Disease Biomarker's Program (PDBP) | 314 | 138 | 198 | 142 |
| Parkinson's Progression Markers Initiative (PPMI) | 243 | 110 | 120 | 55 |
| Baylor College of Medicine / University of Maryland | 508 | 59 | 260 | 136 |
| Spanish Parkinson's (IPDGC) part1 | 1198 | 608 | 910 | 723 |
| Spanish Parkinson's (IPDGC) part2 | 1349 | 739 | 1010 | 814 |
| Tubingen Parkinson's Disease cohort | 421 | 227 | 237 | 307 |
| WTCCC PD GWAS (PMID:21044948) | 982 | 2626 | 627 | 2569 |
| Vance (dbGap phs000394) | 447 | 147 | 172 | 151 |

**Supplementary Table 4. Power Calculations for Mendelian Randomization analyses**

| Trait | Analysis type | Variance in trait explained by instruments (R2) | Power (%) to detect the following odds ratios per SD difference in the trait: | | | | | |
| --- | --- | --- | --- | --- | --- | --- | --- | --- |
|  |  |  | 0.85 | 0.90 | 0.95 | 1.05 | 1.10 | 1.15 |
| Single region analyses | Female | 0.014 | 29.3 | 14.9 | 6.5 | 6.2 | 12.9 | 22.9 |
|  | Male | 0.014 | 38.1 | 18.8 | 7.5 | 7.2 | 16.2 | 29.6 |
| LDL-C | Female | 0.146 | 99.5 | 84.2 | 30.3 | 27.8 | 76.5 | 97.6 |
|  | Male | 0.146 | 100 | 93.4 | 39.3 | 36.2 | 88.1 | 99.6 |

Single region analyses are based variance explained in LDL-C by a single, sentinel variant in *PCSK9* reported in one of the previous GWAS of lipids as used in Williams et al. As noted in Williams et al., LDL-C has robust instrument strength as indicated by R2 values for top GWAS hits explaining 14.6% (from results with both sexes included).
Female-specific analysis is based on a sample of 7,384 Parkinson’s disease cases and 20,330 controls, with 1:2.75 case-control ratio. Male-specific analysis is based on a sample of 12,054 Parkinson’s disease cases with 19,336 controls, 1:1.60 case-control ratio. The significance level is set at α = 0.05. We assume that the variance in the trait explained by the genetic instruments is not different in females versus males.

**Supplementary Table 5. Genetic instrumental variables for the standard (genome-wide) sex-specific Mendelian randomization analysis between LDL-C and late-onset PD**

| Female-specific analysis | | | | |  | Male-specific analysis | | | | |
| --- | --- | --- | --- | --- | --- | --- | --- | --- | --- | --- |
| Genetic instrument for LDL-C (GRCh37) | LDL Beta (SE) | LDL effect allele | PD Beta (SE) | PD effect allele |  | Genetic instrument for LDL-C (GRCh37) | LDL Beta (SE) | LDL effect allele | PD Beta (SE) | PD effect allele |
| 1:109818306 | 0.11 (0.00385) | T | 0.0022 (0.0275) | T |  | 1:109818306 | 0.128 (0.00418) | T | 0.0016 (0.0239) | T |
| 1:63025942 | 0.0477 (0.00336) | T | -0.0152 (0.0241) | T |  | 1:63025942 | 0.0307 (0.00366) | T | 0.0267 (0.0212) | T |
| 10:113933886 | -0.0261 (0.00359) | G | -0.0023 (0.0244) | A |  | 11:116648917 | -0.0314 (0.00517) | C | -0.0238 (0.0287) | C |
| 11:116648917 | -0.0811 (0.00472) | C | -0.0645 (0.0326) | C |  | 11:126243952 | 0.0486 (0.00515) | A | -0.0251 (0.0314) | A |
| 11:126243952 | 0.034 (0.00471) | A | 0.0048 (0.0356) | A |  | 11:61569830 | -0.0276 (0.00367) | T | -0.0062 (0.0212) | T |
| 11:61569830 | -0.0348 (0.00337) | T | 0.0168 (0.0239) | T |  | 12:112072424 | -0.0272 (0.00355) | G | -0.0347 (0.0202) | A |
| 12:112072424 | -0.0239 (0.00325) | G | 0.0273 (0.0232) | A |  | 13:32953388 | -0.0203 (0.00349) | C | -0.0135 (0.0198) | T |
| 12:121416650 | 0.0338 (0.00348) | C | -0.0036 (0.0241) | A |  | 16:72108093 | 0.0426 (0.00447) | A | -0.0259 (0.0248) | A |
| 14:24883887 | 0.0195 (0.00321) | A | -0.0277 (0.0224) | A |  | 17:64210580 | 0.0656 (0.0102) | C | 0.0714 (0.0546) | A |
| 16:56993324 | -0.0485 (0.00342) | A | -0.0294 (0.0245) | A |  | 17:7091650 | -0.0209 (0.00366) | C | 0.0043 (0.023) | T |
| 16:72108093 | 0.0564 (0.00409) | A | -0.047 (0.028) | A |  | 19:11202306 | -0.164 (0.00538) | T | 0.0185 (0.03) | T |
| 17:64210580 | 0.0655 (0.00944) | C | -0.0472 (0.0604) | A |  | 19:45422946 | 0.136 (0.00444) | G | 0.0314 (0.0292) | A |
| 19:11202306 | -0.191 (0.00494) | T | -0.0249 (0.034) | T |  | 2:118835841 | -0.038 (0.00627) | A | 0.0069 (0.0381) | A |
| 19:45422946 | 0.196 (0.00405) | G | 0.0348 (0.0331) | A |  | 2:44065090 | -0.08 (0.00722) | A | 0.0321 (0.0413) | A |
| 2:118835841 | -0.0376 (0.00578) | A | -0.0637 (0.0426) | A |  | 3:32533010 | -0.0361 (0.00614) | T | -0.0252 (0.0346) | T |
| 2:44065090 | -0.11 (0.00662) | A | -0.0409 (0.0465) | A |  | 5:156390297 | 0.0291 (0.00363) | C | 0.0042 (0.0212) | T |
| 3:32533010 | -0.0339 (0.00561) | T | 0.0289 (0.0397) | T |  | 6:26093141 | -0.0579 (0.0065) | A | -0.0876 (0.0456) | A |
| 4:3473139 | 0.0192 (0.00325) | G | 0.0284 (0.0227) | A |  | 6:32412435 | 0.0305 (0.00478) | A | 0.075 (0.0284) | A |
| 5:122855416 | 0.0232 (0.00321) | A | 0.0053 (0.0225) | A |  | 8:59388565 | -0.021 (0.00369) | C | 0.0082 (0.0234) | T |
| 5:131744574 | -0.0224 (0.00409) | C | 0.0043 (0.0279) | T |  | 8:9183358 | 0.0471 (0.00608) | G | 0.0164 (0.0362) | A |
| 5:156390297 | 0.0383 (0.00332) | C | 0.0057 (0.0238) | T |  | -- | -- | -- | -- | -- |
| 6:160578860 | 0.0452 (0.00426) | C | -0.0348 (0.0319) | T |  | -- | -- | -- | -- | -- |
| 6:16145325 | -0.0227 (0.00321) | G | -0.0109 (0.0225) | A |  | -- | -- | -- | -- | -- |
| 6:26093141 | -0.0533 (0.00595) | A | -0.0144 (0.0503) | A |  | -- | -- | -- | -- | -- |
| 6:32412435 | 0.0419 (0.00436) | A | 0.104 (0.0317) | A |  | -- | -- | -- | -- | -- |
| 7:25991826 | 0.0308 (0.0044) | C | 0.0172 (0.0305) | T |  | -- | -- | -- | -- | -- |
| 8:59388565 | -0.0372 (0.00339) | C | 0.0244 (0.026) | T |  | -- | -- | -- | -- | -- |
| 8:9183358 | 0.0387 (0.00553) | G | 0.0071 (0.0408) | A |  | -- | -- | -- | -- | -- |

**Supplementary Table 6. Genome-wide (standard) sex-specific Mendelian randomization results** (Estimates for LDL-lowering targets weighted by circulating LDL estimates)

|  | Female-specific analysis | Male-specific analysis |
| --- | --- | --- |
|  | LDL-C (N Genetic variants used as instrument= 28) | LDL-C (N Genetic variants used as instrument= 20) |
| IVW OR (95% CI); p | 1.07 (0.89 – 1.29); p=0.47 | 0.92 (0.75 – 1.13); p=0.45 |
| Weighted median OR (95% CI); p | 1.06 (0.82 – 1.36); p=0.66 | 0.89 (0.70 – 1.14); p=0.37 |
| Simple mode OR (95% CI); p | 1.06 (0.67 – 1.66); p=0.81 | 0.80 (0.49 – 1.31); p=0.39 |
| Weighted mode OR (95% CI); p | 1.00 (0.80 – 1.25); p=0.98 | 0.89 (0.72 – 1.98); p=0.27 |
| MR-Egger OR (95% CI); p | 1.06 (0.79 – 1.42); p=0.69 | 0.85 (0.62 – 1.78); p=0.35 |
| MR-Egger intercept (p-value) | 0.0007 (p=0.94) | 0.006 (p=0.53) |
| I^2^_GX_ statistic | 99.0% | 98.6% |

**References**

1. Benn M, Nordestgaard BG, Frikke-Schmidt R, Tybjaerg-Hansen A. Low LDL cholesterol, PCSK9 and HMGCR genetic variation, and risk of Alzheimer's disease and Parkinson's disease: Mendelian randomisation study. BMJ. 2017 Apr 24;357:j1648.

2. Fang F, Zhan Y, Hammar N, et al. Lipids, Apolipoproteins, and the Risk of Parkinson Disease. Circ Res. 2019 Aug 30;125(6):643-52.

3. Liu G, Shi M, Mosley JD, et al. A Mendelian Randomization Approach Using 3-HMG-Coenzyme-A Reductase Gene Variation to Evaluate the Association of Statin-Induced Low-Density Lipoprotein Cholesterol Lowering With Noncardiovascular Disease Phenotypes. JAMA Netw Open. 2021 Jun 1;4(6):e2112820.

4. Fu X, Wang Y, He X, Li H, Liu H, Zhang X. A systematic review and meta-analysis of serum cholesterol and triglyceride levels in patients with Parkinson's disease. Lipids Health Dis. 2020 May 19;19(1):97.

5. Jiang Z, Xu X, Gu X, et al. Effects of Higher Serum Lipid Levels on the Risk of Parkinson's Disease: A Systematic Review and Meta-Analysis. Front Neurol. 2020;11:597.

6. Scigliano G, Ronchetti G, Girotti F. Plasma cholesterol and Parkinson's disease: is the puzzle only apparent? Mov Disord. 2010 Apr 15;25(5):659-60.

7. Huang X, Chen H, Petrovitch H, Mailman R, Ross W. Reply: Plasma cholesterol and Parkinson's disease: Is the puzzle only apparent? Mov Disord. 2010 Jan 15;25(1):137.

8. Skrivankova VW, Richmond RC, Woolf BAR, et al. Strengthening the reporting of observational studies in epidemiology using mendelian randomisation (STROBE-MR): explanation and elaboration. BMJ. 2021 Oct 26;375:n2233.

9. Skrivankova VW, Richmond RC, Woolf BAR, et al. Strengthening the Reporting of Observational Studies in Epidemiology Using Mendelian Randomization: The STROBE-MR Statement. JAMA. 2021 Oct 26;326(16):1614-21.

10. Blauwendraat C, Iwaki H, Makarious MB, et al. Investigation of Autosomal Genetic Sex Differences in Parkinson's Disease. Ann Neurol. 2021 Jul;90(1):35-42.

11. Le Guen Y, Napolioni V, Belloy ME, et al. Common X-Chromosome Variants Are Associated with Parkinson Disease Risk. Ann Neurol. 2021 Jul;90(1):22-34.

12. Gillies GE, Pienaar IS, Vohra S, Qamhawi Z. Sex differences in Parkinson's disease. Front Neuroendocrinol. 2014 Aug;35(3):370-84.

13. Taylor KC, Carty CL, Dumitrescu L, et al. Investigation of gene-by-sex interactions for lipid traits in diverse populations from the population architecture using genomics and epidemiology study. BMC Genet. 2013 May 1;14:33.

14. Van PL, Bakalov VK, Bondy CA. Monosomy for the X-chromosome is associated with an atherogenic lipid profile. J Clin Endocrinol Metab. 2006 Aug;91(8):2867-70.

15. Aksglaede L, Molgaard C, Skakkebaek NE, Juul A. Normal bone mineral content but unfavourable muscle/fat ratio in Klinefelter syndrome. Arch Dis Child. 2008 Jan;93(1):30-4.

16. Bojesen A, Kristensen K, Birkebaek NH, et al. The metabolic syndrome is frequent in Klinefelter's syndrome and is associated with abdominal obesity and hypogonadism. Diabetes Care. 2006 Jul;29(7):1591-8.

17. Chen X, McClusky R, Chen J, et al. The number of x chromosomes causes sex differences in adiposity in mice. PLoS Genet. 2012;8(5):e1002709.

18. Charchar FJ, Tomaszewski M, Lacka B, et al. Association of the human Y chromosome with cholesterol levels in the general population. Arterioscler Thromb Vasc Biol. 2004 Feb;24(2):308-12.

19. Zore T, Palafox M, Reue K. Sex differences in obesity, lipid metabolism, and inflammation-A role for the sex chromosomes? Mol Metab. 2018 Sep;15:35-44.

20. Gershoni M, Pietrokovski S. The landscape of sex-differential transcriptome and its consequent selection in human adults. BMC Biol. 2017 Feb 7;15(1):7.

21. Yang X, Schadt EE, Wang S, et al. Tissue-specific expression and regulation of sexually dimorphic genes in mice. Genome Res. 2006 Aug;16(8):995-1004.

22. van Nas A, Guhathakurta D, Wang SS, et al. Elucidating the role of gonadal hormones in sexually dimorphic gene coexpression networks. Endocrinology. 2009 Mar;150(3):1235-49.

23. Wijchers PJ, Yandim C, Panousopoulou E, et al. Sexual dimorphism in mammalian autosomal gene regulation is determined not only by Sry but by sex chromosome complement as well. Dev Cell. 2010 Sep 14;19(3):477-84.

24. Pohjalainen T, Rinne JO, Nagren K, Syvalahti E, Hietala J. Sex differences in the striatal dopamine D2 receptor binding characteristics in vivo. Am J Psychiatry. 1998 Jun;155(6):768-73.

25. Lavalaye J, Booij J, Reneman L, Habraken JB, van Royen EA. Effect of age and gender on dopamine transporter imaging with [123I]FP-CIT SPET in healthy volunteers. Eur J Nucl Med. 2000 Jul;27(7):867-9.

26. Mozley LH, Gur RC, Mozley PD, Gur RE. Striatal dopamine transporters and cognitive functioning in healthy men and women. Am J Psychiatry. 2001 Sep;158(9):1492-9.

27. Laakso A, Vilkman H, Bergman J, et al. Sex differences in striatal presynaptic dopamine synthesis capacity in healthy subjects. Biol Psychiatry. 2002 Oct 1;52(7):759-63.

28. Cantuti-Castelvetri I, Keller-McGandy C, Bouzou B, et al. Effects of gender on nigral gene expression and parkinson disease. Neurobiol Dis. 2007 Jun;26(3):606-14.

29. Cerri S, Mus L, Blandini F. Parkinson's Disease in Women and Men: What's the Difference? J Parkinsons Dis. 2019;9(3):501-15.

30. McCarthy MM. Estradiol and the developing brain. Physiol Rev. 2008 Jan;88(1):91-124.

31. Kuo J, Hamid N, Bondar G, Dewing P, Clarkson J, Micevych P. Sex differences in hypothalamic astrocyte response to estradiol stimulation. Biol Sex Differ. 2010 Nov 22;1(1):7.

32. Vegeto E, Benedusi V, Maggi A. Estrogen anti-inflammatory activity in brain: a therapeutic opportunity for menopause and neurodegenerative diseases. Front Neuroendocrinol. 2008 Oct;29(4):507-19.

33. Azcoitia I, Santos-Galindo M, Arevalo MA, Garcia-Segura LM. Role of astroglia in the neuroplastic and neuroprotective actions of estradiol. Eur J Neurosci. 2010 Dec;32(12):1995-2002.

34. Ciesielska A, Joniec I, Kurkowska-Jastrzebska I, et al. The impact of age and gender on the striatal astrocytes activation in murine model of Parkinson's disease. Inflamm Res. 2009 Nov;58(11):747-53.

35. Villa A, Gelosa P, Castiglioni L, et al. Sex-Specific Features of Microglia from Adult Mice. Cell Rep. 2018 Jun 19;23(12):3501-11.

36. Hanamsagar R, Alter MD, Block CS, Sullivan H, Bolton JL, Bilbo SD. Generation of a microglial developmental index in mice and in humans reveals a sex difference in maturation and immune reactivity. Glia. 2017 Sep;65(9):1504-20.

37. Arevalo MA, Santos-Galindo M, Acaz-Fonseca E, Azcoitia I, Garcia-Segura LM. Gonadal hormones and the control of reactive gliosis. Horm Behav. 2013 Feb;63(2):216-21.

38. Morale MC, Serra PA, L'Episcopo F, et al. Estrogen, neuroinflammation and neuroprotection in Parkinson's disease: glia dictates resistance versus vulnerability to neurodegeneration. Neuroscience. 2006;138(3):869-78.

39. Miletic J, Drakulic D, Pejic S, et al. Prooxidant-antioxidant balance, advanced oxidation protein products and lipid peroxidation in Serbian patients with Parkinson's disease. Int J Neurosci. 2018 Jul;128(7):600-7.

40. Gaignard P, Savouroux S, Liere P, et al. Effect of Sex Differences on Brain Mitochondrial Function and Its Suppression by Ovariectomy and in Aged Mice. Endocrinology. 2015 Aug;156(8):2893-904.

41. Guevara R, Gianotti M, Oliver J, Roca P. Age and sex-related changes in rat brain mitochondrial oxidative status. Exp Gerontol. 2011 Nov;46(11):923-8.

42. Kim HJ, Magrane J, Starkov AA, Manfredi G. The mitochondrial calcium regulator cyclophilin D is an essential component of oestrogen-mediated neuroprotection in amyotrophic lateral sclerosis. Brain. 2012 Sep;135(Pt 9):2865-74.

43. Nakano M, Imamura H, Sasaoka N, et al. ATP Maintenance via Two Types of ATP Regulators Mitigates Pathological Phenotypes in Mouse Models of Parkinson's Disease. EBioMedicine. 2017 Aug;22:225-41.

44. Xing Y, Sapuan A, Dineen RA, Auer DP. Life span pigmentation changes of the substantia nigra detected by neuromelanin-sensitive MRI. Mov Disord. 2018 Nov;33(11):1792-9.

45. Wang LF, Yokoyama KK, Chen TY, et al. Male-Specific Alleviation of Iron-Induced Striatal Injury by Inhibition of Autophagy. PLoS One. 2015;10(7):e0131224.

46. Mariani S, Ventriglia M, Simonelli I, et al. Association between sex, systemic iron variation and probability of Parkinson's disease. Int J Neurosci. 2016;126(4):354-60.

47. Murray HE, Pillai AV, McArthur SR, et al. Dose- and sex-dependent effects of the neurotoxin 6-hydroxydopamine on the nigrostriatal dopaminergic pathway of adult rats: differential actions of estrogen in males and females. Neuroscience. 2003;116(1):213-22.

48. Moroz IA, Rajabi H, Rodaros D, Stewart J. Effects of sex and hormonal status on astrocytic basic fibroblast growth factor-2 and tyrosine hydroxylase immunoreactivity after medial forebrain bundle 6-hydroxydopamine lesions of the midbrain dopamine neurons. Neuroscience. 2003;118(2):463-76.

49. Lewis C, Dluzen DE. Testosterone enhances dopamine depletion by methamphetamine in male, but not female, mice. Neurosci Lett. 2008 Dec 19;448(1):130-3.

50. McArthur S, Murray HE, Dhankot A, Dexter DT, Gillies GE. Striatal susceptibility to a dopaminergic neurotoxin is independent of sex hormone effects on cell survival and DAT expression but is exacerbated by central aromatase inhibition. J Neurochem. 2007 Feb;100(3):678-92.

51. Dewing P, Chiang CW, Sinchak K, et al. Direct regulation of adult brain function by the male-specific factor SRY. Curr Biol. 2006 Feb 21;16(4):415-20.

52. Semchuk KM, Love EJ, Lee RG. Parkinson's disease and exposure to agricultural work and pesticide chemicals. Neurology. 1992 Jul;42(7):1328-35.

53. Bruns J, Jr., Hauser WA. The epidemiology of traumatic brain injury: a review. Epilepsia. 2003;44(s10):2-10.

54. Mantri S, Fullard ME, Duda JE, Morley JF. Physical Activity in Early Parkinson Disease. J Parkinsons Dis. 2018;8(1):107-11.

55. Clark AJ, Ritz B, Prescott E, Rod NH. Psychosocial risk factors, pre-motor symptoms and first-time hospitalization with Parkinson's disease: a prospective cohort study. Eur J Neurol. 2013 Aug;20(8):1113-20.

56. Lupien SJ, McEwen BS, Gunnar MR, Heim C. Effects of stress throughout the lifespan on the brain, behaviour and cognition. Nat Rev Neurosci. 2009 Jun;10(6):434-45.

57. Heinlein CA, Chang C. Androgen receptor (AR) coregulators: an overview. Endocr Rev. 2002 Apr;23(2):175-200.

58. Liao RS, Ma S, Miao L, Li R, Yin Y, Raj GV. Androgen receptor-mediated non-genomic regulation of prostate cancer cell proliferation. Transl Androl Urol. 2013 Sep;2(3):187-96.

59. Kautzky-Willer A, Handisurya A. Metabolic diseases and associated complications: sex and gender matter! Eur J Clin Invest. 2009 Aug;39(8):631-48.

60. Santosa S, Jensen MD. Effects of male hypogonadism on regional adipose tissue fatty acid storage and lipogenic proteins. PLoS One. 2012;7(2):e31473.

61. Palmisano BT, Zhu L, Eckel RH, Stafford JM. Sex differences in lipid and lipoprotein metabolism. Mol Metab. 2018 Sep;15:45-55.

62. Link JC, Reue K. Genetic Basis for Sex Differences in Obesity and Lipid Metabolism. Annu Rev Nutr. 2017 Aug 21;37:225-45.

63. Sugiyama MG, Agellon LB. Sex differences in lipid metabolism and metabolic disease risk. Biochem Cell Biol. 2012 Apr;90(2):124-41.

64. Roselli CE, Horton LE, Resko JA. Distribution and regulation of aromatase activity in the rat hypothalamus and limbic system. Endocrinology. 1985 Dec;117(6):2471-7.

65. Isidori AM, Giannetta E, Greco EA, et al. Effects of testosterone on body composition, bone metabolism and serum lipid profile in middle-aged men: a meta-analysis. Clin Endocrinol (Oxf). 2005 Sep;63(3):280-93.

66. Wang X, Magkos F, Mittendorfer B. Sex differences in lipid and lipoprotein metabolism: it's not just about sex hormones. J Clin Endocrinol Metab. 2011 Apr;96(4):885-93.

67. Whitsel EA, Boyko EJ, Matsumoto AM, Anawalt BD, Siscovick DS. Intramuscular testosterone esters and plasma lipids in hypogonadal men: a meta-analysis. Am J Med. 2001 Sep;111(4):261-9.

68. Elbers JM, Giltay EJ, Teerlink T, et al. Effects of sex steroids on components of the insulin resistance syndrome in transsexual subjects. Clin Endocrinol (Oxf). 2003 May;58(5):562-71.

69. Asscheman H, Gooren LJ, Megens JA, Nauta J, Kloosterboer HJ, Eikelboom F. Serum testosterone level is the major determinant of the male-female differences in serum levels of high-density lipoprotein (HDL) cholesterol and HDL2 cholesterol. Metabolism. 1994 Aug;43(8):935-9.

70. Somboonporn W, Davis S, Seif MW, Bell R. Testosterone for peri- and postmenopausal women. Cochrane Database Syst Rev. 2005 Oct 19(4):CD004509.

71. Meyer MR, Fredette NC, Howard TA, et al. G protein-coupled estrogen receptor protects from atherosclerosis. Sci Rep. 2014 Dec 23;4:7564.

72. Garaulet M, Perez-Llamas F, Baraza JC, et al. Body fat distribution in pre-and post-menopausal women: metabolic and anthropometric variables. J Nutr Health Aging. 2002;6(2):123-6.

73. Rosenbaum M, Pietrobelli A, Vasselli JR, Heymsfield SB, Leibel RL. Sexual dimorphism in circulating leptin concentrations is not accounted for by differences in adipose tissue distribution. Int J Obes Relat Metab Disord. 2001 Sep;25(9):1365-71.

74. Shi H, Seeley RJ, Clegg DJ. Sexual differences in the control of energy homeostasis. Front Neuroendocrinol. 2009 Aug;30(3):396-404.

75. Shimizu H, Shimomura Y, Nakanishi Y, et al. Estrogen increases in vivo leptin production in rats and human subjects. J Endocrinol. 1997 Aug;154(2):285-92.

76. Montague CT, Prins JB, Sanders L, Digby JE, O'Rahilly S. Depot- and sex-specific differences in human leptin mRNA expression: implications for the control of regional fat distribution. Diabetes. 1997 Mar;46(3):342-7.

77. Lapauw B, Ouwens M, t Hart LM, et al. Sex steroids affect triglyceride handling, glucose-dependent insulinotropic polypeptide, and insulin sensitivity: a 1-week randomized clinical trial in healthy young men. Diabetes Care. 2010 Aug;33(8):1831-3.

78. Fraser IS, Jansen RP, Lobo RA, Whitehead MI. Estrogens and progestogens in clinical practice. Churchill Livingstone. 1998.

79. Godsland IF. Effects of postmenopausal hormone replacement therapy on lipid, lipoprotein, and apolipoprotein (a) concentrations: analysis of studies published from 1974-2000. Fertil Steril. 2001 May;75(5):898-915.

80. Walsh BW, Sacks FM. Effects of low dose oral contraceptives on very low density and low density lipoprotein metabolism. J Clin Invest. 1993 May;91(5):2126-32.

81. Duvillard L, Dautin G, Florentin E, Petit JM, Gambert P, Verges B. Changes in apolipoprotein B100-containing lipoprotein metabolism due to an estrogen plus progestin oral contraceptive: a stable isotope kinetic study. J Clin Endocrinol Metab. 2010 May;95(5):2140-6.

82. Furuhashi M, Hotamisligil GS. Fatty acid-binding proteins: role in metabolic diseases and potential as drug targets. Nat Rev Drug Discov. 2008 Jun;7(6):489-503.

83. Maher AC, Akhtar M, Vockley J, Tarnopolsky MA. Women have higher protein content of beta-oxidation enzymes in skeletal muscle than men. PLoS One. 2010 Aug 6;5(8):e12025.

84. Heinzel S, Kasten M, Behnke S, et al. Age- and sex-related heterogeneity in prodromal Parkinson's disease. Mov Disord. 2018 Jul;33(6):1025-7.

85. Wooten GF, Currie LJ, Bovbjerg VE, Lee JK, Patrie J. Are men at greater risk for Parkinson's disease than women? J Neurol Neurosurg Psychiatry. 2004 Apr;75(4):637-9.

86. Haaxma CA, Bloem BR, Borm GF, et al. Gender differences in Parkinson's disease. J Neurol Neurosurg Psychiatry. 2007 Aug;78(8):819-24.

87. Park K, Oeda T, Kohsaka M, Tomita S, Umemura A, Sawada H. Low body mass index and life prognosis in Parkinson's disease. Parkinsonism Relat Disord. 2018 Oct;55:81-5.

88. Kim R, Lee J, Kim Y, et al. Presynaptic striatal dopaminergic depletion predicts the later development of freezing of gait in de novo Parkinson's disease: An analysis of the PPMI cohort. Parkinsonism Relat Disord. 2018 Jun;51:49-54.

89. Ou R, Liu H, Hou Y, et al. Predictors of camptocormia in patients with Parkinson's disease: A prospective study from southwest China. Parkinsonism Relat Disord. 2018 Jul;52:69-75.

90. Baba Y, Putzke JD, Whaley NR, Wszolek ZK, Uitti RJ. Gender and the Parkinson's disease phenotype. J Neurol. 2005 Oct;252(10):1201-5.

91. Yoritaka A, Ohizumi H, Tanaka S, Hattori N. Parkinson's disease with and without REM sleep behaviour disorder: are there any clinical differences? Eur Neurol. 2009;61(3):164-70.

92. Ozekmekci S, Apaydin H, Kilic E. Clinical features of 35 patients with Parkinson's disease displaying REM behavior disorder. Clin Neurol Neurosurg. 2005 Jun;107(4):306-9.

93. Martinez-Martin P, Falup Pecurariu C, Odin P, et al. Gender-related differences in the burden of non-motor symptoms in Parkinson's disease. J Neurol. 2012 Aug;259(8):1639-47.

94. Clark US, Neargarder S, Cronin-Golomb A. Specific impairments in the recognition of emotional facial expressions in Parkinson's disease. Neuropsychologia. 2008;46(9):2300-9.

95. Cholerton B, Johnson CO, Fish B, et al. Sex differences in progression to mild cognitive impairment and dementia in Parkinson's disease. Parkinsonism Relat Disord. 2018 May;50:29-36.

96. Szewczyk-Krolikowski K, Tomlinson P, Nithi K, et al. The influence of age and gender on motor and non-motor features of early Parkinson's disease: initial findings from the Oxford Parkinson Disease Center (OPDC) discovery cohort. Parkinsonism Relat Disord. 2014 Jan;20(1):99-105.

97. Lin SJ, Baumeister TR, Garg S, McKeown MJ. Cognitive Profiles and Hub Vulnerability in Parkinson's Disease. Front Neurol. 2018;9:482.

98. Weintraub D, Koester J, Potenza MN, et al. Impulse control disorders in Parkinson disease: a cross-sectional study of 3090 patients. Arch Neurol. 2010 May;67(5):589-95.

99. Bhattacharjee S. Impulse control disorders in Parkinson's disease: Review of pathophysiology, epidemiology, clinical features, management, and future challenges. Neurol India. 2018 Jul-Aug;66(4):967-75.

100. Ophey A, Eggers C, Dano R, Timmermann L, Kalbe E. Health-Related Quality of Life Subdomains in Patients with Parkinson's Disease: The Role of Gender. Parkinsons Dis. 2018;2018:6532320.

101. Buczak-Stec EW, Konig HH, Hajek A. Impact of Incident Parkinson's Disease on Satisfaction With Life. Front Neurol. 2018;9:589.

102. Dahodwala N, Shah K, He Y, et al. Sex disparities in access to caregiving in Parkinson disease. Neurology. 2018 Jan 2;90(1):e48-e54.

103. Larsson V, Torisson G, Londos E. Relative survival in patients with dementia with Lewy bodies and Parkinson's disease dementia. PLoS One. 2018;13(8):e0202044.

104. Colombo D, Abbruzzese G, Antonini A, et al. The "gender factor" in wearing-off among patients with Parkinson's disease: a post hoc analysis of DEEP study. ScientificWorldJournal. 2015;2015:787451.

105. Nienstedt JC, Bihler M, Niessen A, et al. Predictive clinical factors for penetration and aspiration in Parkinson's disease. Neurogastroenterol Motil. 2019 Mar;31(3):e13524.

106. Lemieux S, Prud'homme D, Bouchard C, Tremblay A, Despres JP. Sex differences in the relation of visceral adipose tissue accumulation to total body fatness. Am J Clin Nutr. 1993 Oct;58(4):463-7.

107. Abbott RD, Garrison RJ, Wilson PW, et al. Joint distribution of lipoprotein cholesterol classes. The Framingham study. Arteriosclerosis. 1983 May-Jun;3(3):260-72.

108. Magkos F, Mohammed BS, Mittendorfer B. Effect of obesity on the plasma lipoprotein subclass profile in normoglycemic and normolipidemic men and women. Int J Obes (Lond). 2008 Nov;32(11):1655-64.

109. Freedman DS, Otvos JD, Jeyarajah EJ, et al. Sex and age differences in lipoprotein subclasses measured by nuclear magnetic resonance spectroscopy: the Framingham Study. Clin Chem. 2004 Jul;50(7):1189-200.

110. Leppert MH, Burke JF, Lisabeth LD, et al. Systematic Review of Sex Differences in Ischemic Strokes Among Young Adults: Are Young Women Disproportionately at Risk? Stroke. 2022 Feb;53(2):319-27.

111. Maas AH, Appelman YE. Gender differences in coronary heart disease. Neth Heart J. 2010 Dec;18(12):598-602.

112. Benjamin EJ, Blaha MJ, Chiuve SE, et al. Heart Disease and Stroke Statistics-2017 Update: A Report From the American Heart Association. Circulation. 2017 Mar 7;135(10):e146-e603.

113. Toth MJ, Tchernof A, Sites CK, Poehlman ET. Menopause-related changes in body fat distribution. Ann N Y Acad Sci. 2000 May;904:502-6.

114. Shulman LM, Bhat V. Gender disparities in Parkinson's disease. Expert Rev Neurother. 2006 Mar;6(3):407-16.

115. Kumagai T, Nagayama H, Ota T, Nishiyama Y, Mishina M, Ueda M. Sex differences in the pharmacokinetics of levodopa in elderly patients with Parkinson disease. Clin Neuropharmacol. 2014 Nov-Dec;37(6):173-6.

116. Goudreau JL, Maraganore DM, Farrer MJ, et al. Case-control study of dopamine transporter-1, monoamine oxidase-B, and catechol-O-methyl transferase polymorphisms in Parkinson's disease. Mov Disord. 2002 Nov;17(6):1305-11.

117. Lyons KE, Hubble JP, Troster AI, Pahwa R, Koller WC. Gender differences in Parkinson's disease. Clin Neuropharmacol. 1998 Mar-Apr;21(2):118-21.

118. Zappia M, Crescibene L, Arabia G, et al. Body weight influences pharmacokinetics of levodopa in Parkinson's disease. Clin Neuropharmacol. 2002 Mar-Apr;25(2):79-82.

119. Tsang KL, Ho SL, Lo SK. Estrogen improves motor disability in parkinsonian postmenopausal women with motor fluctuations. Neurology. 2000 Jun 27;54(12):2292-8.

120. Nicoletti A, Arabia G, Pugliese P, et al. Hormonal replacement therapy in women with Parkinson disease and levodopa-induced dyskinesia: a crossover trial. Clin Neuropharmacol. 2007 Sep-Oct;30(5):276-80.

121. Pavanello CM, G. Considering gender in prescribing statins: what do physicians need to know? Clinical Lipidology. 2017;10(6):499-512.

122. Nanna MG, Wang TY, Xiang Q, et al. Sex Differences in the Use of Statins in Community Practice. Circ Cardiovasc Qual Outcomes. 2019 Aug;12(8):e005562.

123. Skilving I, Eriksson M, Rane A, Ovesjo ML. Statin-induced myopathy in a usual care setting-a prospective observational study of gender differences. Eur J Clin Pharmacol. 2016 Oct;72(10):1171-6.
